# Age-related dysregulation of CXCL9/10 in monocytes is linked to impaired innate immune responses in a mouse model of *Staphylococcus aureus* osteomyelitis

**DOI:** 10.1007/s00018-024-05311-2

**Published:** 2024-07-13

**Authors:** Yihuang Lin, Mankai Yang, Chubin Cheng, Jichang Wu, Bin Yu, Xianrong Zhang

**Affiliations:** 1 Division of Orthopaedics and Traumatology, Department of Orthopaedics, Nanfang Hospital, Southern Medical University, No. 1838 North of Guangzhou Avenue, Guangzhou, Guangdong Province 510515 China; 2 Guangdong Provincial Key Laboratory of Bone and Cartilage Regenerative Medicine, Nanfang Hospital, Southern Medical University, Guangzhou, Guangdong Province 510515 China; 3 Department of Orthopaedics, Zhangzhou Affiliated Hospital of Fujian Medical University, Zhangzhou, 363000 China

**Keywords:** *Staphylococcus aureus*, Osteomyelitis, Monocytes, CXCL9, CXCL10, Innate immune

## Abstract

**Background:**

Age-associated impairments in innate immunity are believed to be a causative factor responsible for severe pathogenesis of *Staphylococcus aureus* (*S. aureus*) infection in the bone tissue. However, the basis for age-associated decline in innate immune response upon *S. aureus* infection remains poorly understood.

**Results:**

Our transcriptional data (GEO: GSE166522) from a mouse model of *S. aureus* osteomyelitis show up-regulated CXCL9 and CXCL10 (CXCL9/10), which is further confirmed in vitro and in vivo by the present study. Notably, monocytes are a main source for CXCL9/10 production in bone marrow upon *S. aureus* challenge, but this response declines in middle-aged mice. Interestingly, conditional medium of bone marrow monocytes from middle-aged mice has a strikingly decreased effect on bactericidal functions of neutrophils and macrophages compares with that from young mice. We further show that activation of CXCL9/10-CXCR3 axis between monocytes and macrophages/neutrophils promotes the bactericidal function of the cells, whereas blocking the axis impairs such function. Importantly, treatment with either exogenous CXCL9 or CXCL10 in a middle-aged mice model enhances, while pharmacological inhibition of CXCR3 in young mice model impairs, bacterial clearance and bone marrow structure.

**Conclusions:**

These findings demonstrate that bone marrow monocytes act as a critical promotor of innate immune response via the CXLCL9/10-CXCR3 axis upon *S. aureus* infection, and that the increased susceptibility to *S. aureus* infection in skeleton in an aged host may be largely attributable to the declined induction of CXCR9/10 in monocytes.

**Supplementary Information:**

The online version contains supplementary material available at 10.1007/s00018-024-05311-2.

## Background

*Staphylococcus aureus* (*S. aureus*) is the main causative pathogen of osteomyelitis in long bones and vertebrae [1, 2]. Epidemiological data have shown that the incidence of osteomyelitis has increased over the past decade [1, 3, 4]. One of the major risk factors for pyrogenic osteomyelitis is age, especially older than 65 years [4, 5]. Notably, population-based cohort studies indicate that chronic osteomyelitis increases the rate of fragility fracture [6] and the risk of long-term mortality in the elderly individuals [7]. It is therefore vital to understand the pathogenic mechanisms of osteomyelitis in aged bone to uncover therapeutic strategies that bolster antibacterial immunity.

It is well known that greater susceptibility to and severity of infection in aged individuals are associated with compromised function of immune system [8, 9]. As the first-line defense against bacterial invasion, neutrophils, monocytes and macrophages play a decisive role in clearance of pathogens and control of pathogenesis [10]. Extensive evidence has described the declined function of neutrophils and macrophages from older subjects or animals, such as inaccurate migration of neutrophils from older adults [11] and in inflamed tissues in aged mice [12], diminished phagocytosis of macrophages in aged mice [13, 14] and decreased bactericidal function of aged mice macrophages [15, 16], contributing to the inability to control pathogen growth and subsequent tissue damage.

Dysfunction of innate immunity with aging is associated with altered biological processes. Several recent studies have shown that monocytes from elderly individuals have increased inflammatory transcriptional programs and senescence-associated secretory phenotype, and a shift of their subset composition under the basal state [17–19], and that from aged mice have altered subpopulation and expression of signature genes [18]. In addition, the interactions among immune cells in the local immune microenvironment may change with aging as well, for example, monocytes/macrophages in aged tissue stimulate recruitment of neutrophils to aggravate tissue injury [20]. However, how these changes shape the host anti-bacterial immunity is still not fully understood.

In this study, we uncovered a previously uncharacterized role of monocyte-originated CXCL9/10 in regulation of bactericidal function of neutrophils and macrophages in vitro and at an acute stage of *S. aureus* osteomyelitis in mice. We also unraveled that the reduced bacterial clearance and exacerbated injury in bone marrow of middle-aged mice attributed to the declined production of CXCL9/10 in monocytes upon *S. aureus* challenge. Our results suggest that targeting CXCL9/10-CXCR3 axis could be a promising therapeutic approach to treatment of age-associated *S. aureus* osteomyelitis.

## Methods

### Bacterial preparation

The *S. aureus* strain was isolated from a patient with chronic tibial osteomyelitis the pathogen of which was identified as methicillin-sensitive *S. aureus*. For resuscitation of *S. aureus*, 10 µl of frozen sample was inoculated to 3 ml tryptic soy broth (TSB) medium and shaken at 180 rpm in an incubator shaker at 37 ℃. After 16–18 h of incubation, 1 ml of bacterial suspension was collected and centrifuged at 2500 g for 5 min. The *S. aureus* pellet was resuspended in 1 ml PBS and centrifuged again before washes repeated for three times. The concentration of *S. aureus* was adjusted to an optical density (OD) of 0.5 at 600 nm, approximately equal to 1 × 10^8^ colony forming unit (CFU) per milliliter.

### Implant-associated osteomyelitis mouse models and treatments

All animal experimental protocols were approved by the Animal Care and Use Committee of Nanfang Hospital, Southern Medical University. C57BL/6 male mice were obtained from the Animal Center of Southern Medical University and housed under a 12-h light-dark cycle, 24 ± 2 ℃ room temperature, and ad libitum access to water and food. Young (8-week-old) and middle-aged (10-month-old) mice were used to establish implant-associated *S. aureus* osteomyelitis models, as we described previously [21]. By day 3 post-infection, infected femurs were harvested and either subjected to quantify bacterial load in bone tissue or processed to histological staining.

For the CXCR3 blockade treatment in vivo, 8-week-old mice were injected subcutaneously with AMG487 (5 mg/kg, #HY-15,319, MedChemExpress, USA) 6 h after infection, and then injected again every 12 h for a total of 6 times, while the control group was injected with the same volume of vehicle (1% DMSO). To activate CXCR3 signaling in middle-aged mice with *S. aureus* osteomyelitis, 10-month-old mice were injected with 100 ng recombinant mouse CXCL9 protein (#C600269, BBI Life Science, Shanghai, China) or recombinant mouse CXCL10 protein (#HY-P722, MedChemExpress, USA) into the bone marrow cavity using a microsyringe, while the control group was injected with the same volume of PBS. Subsequently, bacterial inoculation and placement of internal implants were performed. By day 3 post-infection, infected femurs were harvested and processed to histological staining.

### Quantification of bacterial load in bone tissue

To quantify the colony number of *S. aureus* in infected femurs, mice were sacrificed by day 3 after infection. The femurs were isolated and then crushed with a tissue homogenizer. The homogenized femurs were weighed and made into homogenate at a weight to volume ratio of 0.1 g/ml in phosphate buffer saline (PBS). The homogenate was subjected to serial dilutions and incubated on TSB agar plates at 37 ℃ for 24 h.

### Histological staining

The operated femurs were collected by day 3 after operation, then fixed with 4% paraformaldehyde for 24 h, decalcified with 0.5 M Ethylenediaminetetraacetic acid (EDTA) for 7 days, and finally embedded in paraffin. Paraffin sections at 4 μm were collected for hematoxylin and eosin (H&E) or immunohistochemical and immunofluorescence staining.

For immunofluorescence staining of *S. aureus*, after deparaffinization and rehydration of paraffin sections, antigen retrieval was performed with Tris-EDTA solution (pH = 9) at 70 ℃ for 30 min, followed by blocking with 10% goat serum at room temperature for 1 h. Sections were incubated with the rabbit anti-*S. aureus* antibody (#Ab20920, Abcam, USA) at 4 ℃ overnight. iFluor 594-conjugated goat anti-rabbit IgG (#HA1122, Huabio, Hangzhou, China) was used as a secondary antibody. Nuclei were counterstained with DAPI (#E607303-0002; BBI Life Science, Shanghai, China). Sections were observed under a BX53 microscope (Olympus, Tokyo, Japan) and analyzed by Image J (v1.8.0).

For immunohistochemical analysis, after deparaffinization and rehydration of paraffin sections, antigen retrieval was performed with Tris-EATA (pH = 9) for 3 h at 70 ℃ and endogenous peroxidase was inactivated by 3% H_2_O_2_ for 15 min. After being blocked with 10% goat serum for 1 h at room temperature, sections were incubated with the rabbit anti-Phospho- mixed-lineage kinase domain-like (MLKL) antibody (ET1705-51, Huabio, Hangzhou, China) or anti-CXCL9 antibody (#ABS124268, Absin, Shanghai, China) or anti-CXCL10 antibody (#DF6417, Affinity, Liyang, China) overnight at 4 °C. Next, horseradish peroxidase (HRP)-conjugated goat anti-rabbit IgG antibody (HA1001, Huabio, Hangzhou, China) was used as a secondary antibody and incubated for 1 h at room temperature. Then, peroxidase activity was revealed by 3,3-N-Diaminobenzidine tertrahydrochloride (DAB) substrate kit (#ZLI-9017, ZSGB-BIO, Beijing, China) according to manufacturer’s protocol. Finally, the nuclei were counterstained with hematoxylin (#H-3404-100, Vector). Sections were observed under a microscope (Eclipse Ci-L plus, Nikon, Tokyo, Japan) and analyzed by Image J (v1.8.0).

### Flow cytometry

The infected mice femurs and control ones were harvested on day 3 after surgery. The bone marrow cells were flushed out and collected in RPMI 1640 medium. After being filtered through a 70 μm strainer, whole bone marrow cells were counted after lysis of red blood cells. To block intracellular cytokine secretion, cells were treated with Brefeldin A (#GC17683, GLPBIO, USA) at a concentration of 10 µg/ml and incubated at 37 ℃ for 2 h. Next, cells were incubated with anti-mouse CD16/32 (#101,319, Biolegend, USA) to block non-specific staining on ice, followed by incubation with primary antibodies, including BV421-conjugated anti-mouse CD11b antibody (#101,235, Biolegend, USA), BV510-conjugated anti-mouse Ly6C antibody (#128,033, Biolegend, USA), PerCP/Cy5.5-conjugated anti-mouse Ly6G antibody (#127,615, Biolegend, USA), APC/CY7-conjugated anti-mouse F4/80 antibody (#123,117, Biolegend, USA), FITC anti-mouse CD3 (#100,203, Biolegend, USA), APC anti-mouse CD8a (#100,711, Biolegend, USA), or PE anti-mouse CD4 (#100,407, Biolegend, USA). Finally, samples were detected on the flow cytometer (LSRFortessa X-20, BD Bioscience, USA) and analyzed by FlowJo (V10).

### Bioinformatics analysis

The DESeq2 R language (1.16.1) was used to analyze our previous transcriptome data (GEO: GSE166522) of bone tissue from mice (10–12 weeks old) with *S. aureus* osteomyelitis and control ones by day 3 after operation. Differential expressed genes (DEGs) were determined if the gene | log2FoldChange | > 1 and adjusted *p* values < 0.05. ClusterProfiler R software (3.6.2) was used to perform Gene Ontology (GO) enrichment analysis of differential genes, and adjusted *p* value < 0.05 was considered as significant enrichment.

### Isolation and identification of monocytes, neutrophils and bone marrow derived macrophages (BMDMs)

After collection of bone marrow single cell suspension, bone marrow neutrophils and monocytes were isolated by density gradient centrifugation. Briefly, 3 ml of Percoll working solution and suspended single cell solution with concentrations of 1.09, 1.077 and 1.043 g/ml were successively added into a 15 ml centrifuge tube, and centrifuged at room temperature for 35 min under slow acceleration condition. The neutrophils were extracted at the interface of 1.09 g/ml and 1.077 g/ml. Monocytes-lymphocytes were extracted at the interface of 1.077 g/ml and 1.043 g/ml and then centrifuged at 1.067 g/ml Percoll working solution to further separate the upper layer of monocytes. The collected neutrophils and monocytes were washed twice with PBS and resuspended in RPMI 1640 containing 10% FBS before the next experiment.

For BMDMs isolation, bone marrow cells from 8-week-old or 10-month-old mice were flushed out with PRMI 1640 medium, centrifuged, resuspended in macrophages growth medium (RPMI 1640, 10% FBS, 20% L929 cell-conditioned medium, and 1% penicillin/streptomycin) at 2 × 10^6^ cells /ml, and then seeded in cell culture dishes. After 7 days, bone marrow cells were differentiated into mature BMDMs.

The purity of isolated neutrophils, monocytes, and BMDMs from mice bone marrow were analyzed by detecting expression of cell surface markers and evaluating the proportion of CD11b^+^Ly6G^+^, CD11b^+^Ly6C^+^, and CD11b^+^F4/80^+^ cell populations, respectively. For freshly isolated neutrophils and monocytes, cells were incubated with anti-CD16/CD32 antibodies (#101,319, Biolegend, USA) in 0.5% BSA/PBS buffer on ice for 10 min, and then stained with a combination of FITC anti-CD11b (#101,205, Biolegend, USA) plus BV510 anti-Ly6G (#127,633, Biolegend, USA), and a combination of FITC anti-CD11b (#101,205, Biolegend, USA) plus PerCP anti-Ly6C (#128,027, Biolegend, USA), respectively, at room temperature for 30 min. For evaluating the purity of primary BMDMs, after 7 days differentiation, BMDMs were trypsinized with 0.25% Trypsin-EDTA, centrifugated and washed twice with PBS, then labeled with FITC anti-CD11b (#101,205, Biolegend, USA) and APC/Fire 750 anti-F4/80 (#123,151, Biolegend, USA) at room temperature for 30 min. Unstained cells were used as the negative control. After being washed twice, cells were resuspended in 0.5%BSA/PBS buffer and immediately detected using a Beckman CytoFLEX flow cytometer (A00-1-1102).

### Cell treatment and conditional medium (CM) experiment

BMDMs (2 × 10^6^ cells/well) were seeded in 6-well plates and stimulated with *S. aureus* in macrophages growth medium without antibiotics at various multiplicity of infection (MOI) (0, 2, 10 and 50) for 1 h. After 1% penicillin/streptomycin was added to the co-culture system to inhibit bacterial overgrowth, the culture was continued for 5 h. The medium was discarded and washed twice with PBS before finally TRIZOL (#AG21102, Accurate Biology, Changsha, China) was added to the well for total RNA extraction.

2 × 10^6^ cells /ml monocytes or neutrophils suspension was seeded per well in 12-well plates. 1 ml. *S. aureus* at various MOI (0, 2, 10 and 50) was used to stimulate cells for 1 h, and then 1% penicillin/streptomycin was added to the co-culture system to inhibit bacterial overgrowth. The culture was continued for 5 h. After centrifugation at 300 g for 5 min, the pellet of monocytes or neutrophils was resuspended in TRIZOL for total RNA extraction. For CM preparation, the supernatant of monocytes was collected into a new centrifuge tube, centrifuged at 2500 g for 5 min to remove most of the bacteria, and finally filtered through a 0.22 μm filter. CM of monocytes treated with *S. aureus* (MOI = 10, CM Mono-*S. aureus*) and CM of monocytes treated with PBS (MOI = 0, CM Mono-vehicle) were reserved for next experiments.

For siRNA knockdown experiment, monocytes from 8-week-old mice were isolated as mentioned above, and resuspended in Opti-MEM medium (#31985070, GIBCO). Silencing sequences were transferred into cells with transfection reagents (#40806ES02, Yeasen, Shanghai, China) according to the manufacturer’s instructions and cultured for 24 h. After cells were harvested, the viable cell concentration was readjusted to 2 × 10^6^ cells /ml. 1 ml per well was seeded into 12-well plates. Afterwards, *S. aureus* stimulation procedures as well as cellular RNA collection and conditioned medium collection were performed as described above. Three fragments each for CXCL9 and CXCL10 were screened for evaluating their knock-down efficiency. The sequences of siRNA fragments used for further experiments were as follows: si-CXCL9, 5’GUCGUCGUUCAAGGAAGACUAdTdT3’ and 5’UAGUCUUCCUUGAACGACGACdTdT3’; si-CXCL10, 5’CGGAAUCUAAGACCAUCAAdTdT3’ and 5’UUGAUGGGCUUAGAUUCCGdTdT3’; si-negative control (si-NC), 5’UUCUCCGAACGUGUCACGUdTdT3’ and 5’ ACGUGACACGUUCGGAGAAdTdT3’.

### Quantitative real-time PCR (qPCR)

Bone tissue or cell total RNA was reverse transcribed into cDNA using a reverse transcription kit (11141ES60, Yeasen, Shanghai, China), and qPCR was performed using SYBR Green (11202ES08, Yeasen, Shanghai, China) on QuantStudio6 (Applied Biosystems, USA) according to the manufacturer’s protocol. The primer sequences were obtained from the PrimerBank database [22] as follows: CXCL9, forward: 5’GGAGTTCGAGGAACCCTAGTG3’, reverse: 5’GGGATTTGTAGTGGATCGTGC3’; IL-1β, forward: 5’TCCTGTGTAATGAAAGACGGC3’, reverse: 5’ACTCCACTTTGCTCTTGACTTC3’; CXCL10, forward: 5’CCAAGTGCTGCCGTCATTTTC3’, reverse: 5’GGCTCGCAGGGATGATTTCAA3’; GAPDH, forward: 5’TGTCGTGGAGTCTACTGGTG3’, reverse: 5’GCATTGCTGACAATCTTGAG3’.

### Analysis of live/dead viability and ROS production of isolated neutrophils

Given that neutrophils in ex vivo culture have a short life-time [23], we evaluated the viability of neutrophils after *S. aureus* infection. The isolated neutrophils were cultured in growth medium (RPMI 1640 containing 10% FBS) for 4 h, followed by 30 min of *S. aureus* challenge at MOI of 10. After centrifugation at 300 g for 5 min, the cell pellet was washed twice with PBS, then the cell viability was tested using a calcein/propidium iodide (PI) viability/cytotoxicity assay kit (#C2015M, Beyotime, Shanghai, China). Cells were incubated in buffer containing calcein and PI for 30 min at 37℃ in the dark following the manufacturer’s protocol. After being washed twice with serum-free medium, cells were resuspended in 0.5 ml flow cytometry buffer and immediately detected using a Beckman CytoFLEX flow cytometer (A00-1-1102).

The activated neutrophils destroy bacteria by producing reactive oxygen species (ROS), neutrophil extracellular traps (NETs) formation and release of antimicrobial proteinase [23]. We therefore evaluated the responsivity of neutrophils by determine the levels of ROS production in response to *S. aureus* challenge. After 4 h of culture and 30 min of *S. aureus* challenge as aforementioned, cells were incubated with a staining solution containing 10 µM dihydroethidium (DHE) (#D1004, UEland, Suzhou, China) at 37℃ in the dark for 30 min. Subsequently, cells were washed twice with serum-free medium and immediately detected using a Beckman CytoFLEX flow cytometer (A00-1-1102).

### Analysis of bactericidal activity of neutrophils and macrophages

For the neutrophil bactericidal assay, 1 × 10^6^ neutrophils were stimulated with 500 µl of the medium containing different stimuli for 4 h, washed twice with PBS and resuspended in neutrophil medium. Next, *S. aureus* (MOI = 10) was added and co-cultured for 30 min. After centrifugation at 300 g for 5 min, the cell pellet was washed twice with PBS, and the washing solution was transferred to a new centrifuge tube together with the cell supernatant from the first centrifugation. This solution contained the remaining extracellular bacteria that had not been killed. Cell pellet was lysed with 1 ml of 0.2% Triton X-100 (#T8200, Solarbio) for 15 min at room temperature, thereby releasing residual intracellular bacteria. The remaining intracellular bacteria and the remaining extracellular bacteria were diluted in 10×, 100× and 1000×, and 10 µl of the bacterial solution was inoculated on TSA plates and incubated at 37 ℃ for 20 h.

For macrophage bactericidal assay, 1 × 10^6^ BMDMs were stimulated with 500 µl of the medium containing different stimuli for 4 h, and subsequently co-cultured with *S. aureus* (MOI = 10) for 30 min. After washing twice with PBS, the extracellular bacteria were killed with gentamicin (50 µg/ml) and lysozyme (20 µg/ml) for 30 min. Then the cells were lysed with 0.2% Triton X-100 to release the remaining intracellular bacteria. After 10×, 100× and 1000× dilution, 10 µl of the bacterial solution was inoculated on TSA plates and incubated at 37 ℃ for 20 h.

The medium containing different stimuli mentioned above included: CM of *S. aureus*-challenged monocytes from 8-week-old mice or 10-month-old mice (CM-8 W Mono-*S. aureus*, CM-10 M Mono-*S. aureus*), CM of vehicle-treated monocytes from 8-week-old mice or 10-month-old mice (CM-8 W Mono-Vehicle, CM-10 M Mono-Vehicle), CM-8 W Mono-*S. aureus* pretreated with siRNA fragments targeting CXCL9, or CXCL10, or both CXCL9 and CXCL10, or pretreated with 100 ng/ml of recombinant CXCL9, or recombinant CXCL10, or a combination of CXCL9 and CXCL10. For use of CXCR3 inhibitor, it was pre-stimulated with 500 µM AMG487 for 1 h before addition of CM-8 W Mono-*S. aureus*, recombinant CXCL9 or CXCL10.

### Statistical analysis

GraphPad Prim 9 was used for statistical analysis of the data. If the data were in accordance with normal distribution and homogeneity of variance, the Student’s *t* test was used to compare the statistical differences between the two groups. One-way analysis of variance (One-way ANVOA) with Bonferroni or Dunnett’s T3 *post hoc* test was used for statistical comparison of more than two groups. All data are expressed as mean ± SD. *p* < 0.05 was considered statistically significant.

## Results

### Middle-aged mice have a higher bacterial burden and fewer innate immune cells in the *S. aureus*-infected femurs

**Fig. 1:**
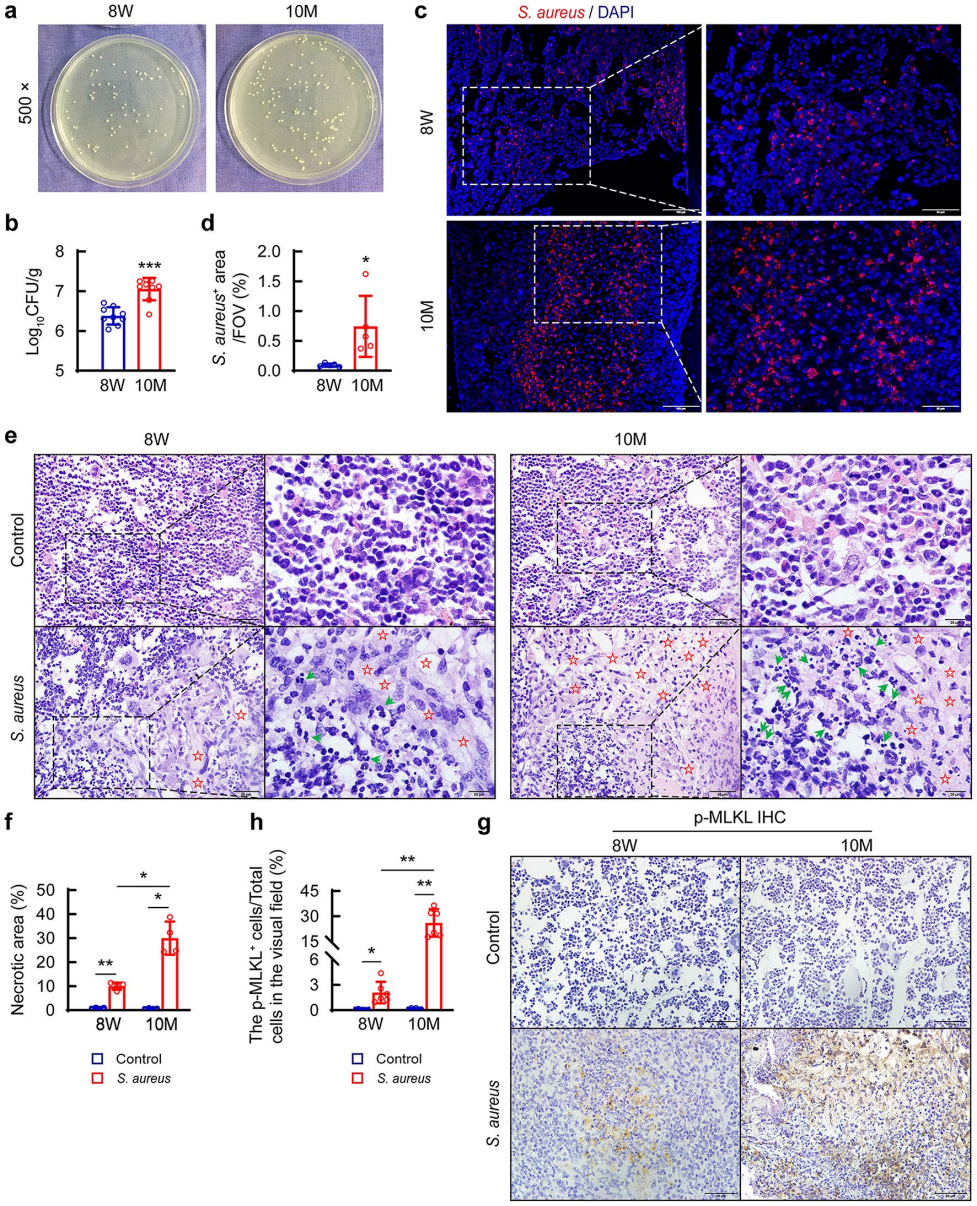
10-month-old mice have higher bacterial burden and less bone destruction in the *S. aureus*-infected femurs. The young (8-week-old) mice and middle-aged (10-month-old) mice were used to establish the model of *S. aureus* osteomyelitis. The right femurs of *S. aureus*-infected and controls were collected for further analysis on the day 3 after surgery. (**a**) Representative images of bacterial colonies on the agar plate and (**b**) quantification of bone tissue bacterial load. *n* = 9/ group. Student’s *t* test, *** *p* < 0.001. (**c**) Representative images of *S. aureus* immunofluorescence staining, with red indicating *S. aureus* positive staining, blue indicating DAPI stained nucleus, and scale bars = 100 μm (left panels) and 50 μm (blowups in the right panels). (**d**) The percentage of *S. aureus*-positive stained area in the bone marrow cavity area. *n* = 5/group. Student’s *t* test, * *p* < 0.05. (**e**) Representative images of hematoxylin and eosin (H&E) staining. The red star indicates the cellular lytic changes, the green arrow nuclear fragmentation. scale bars = 50 μm (left panels) and 20 μm (blowups in the right panels). (**f**) Quantitative analysis of necrotic area in the bone marrow. *n* = 4/ group. One-way ANOVA with Dunnett’s T3 *post hoc* test, *p < 0.05, **p < 0.01. Representative images of immunohistochemistry staining (**g**) and quantitative analysis (**h**) of phosphorylated mixed lineage domain-like (p-MLKL). *n* = 6/ group. One-way ANOVA with Dunnett’s T3 *post hoc* test, * *p* < 0.05, **p < 0.01

To assess the impact of age on host response to *S. aureus* infection in bone, we generated *S. aureus* osteomyelitis in the femurs in young (8-week-old) and middle-aged (10-month-old) mice. Analysis of bacteria load from homogenized bone tissue indicated that there were more *S. aureus* colonies in the infected femurs in 10-month-old mice than in those in 8-week-old mice at day 3 after infection (Fig. 1a and b). Immunofluorescence staining of *S. aureus* showed a strikingly higher number of positively stained bacteria in the bone marrow in 10-month-old mice (Fig. 1c and d). The extensive expansion of *S. aureus* in the bone marrow correlated with massive cellular lytic pathological changes surrounding infectious foci in the infected femurs in 10-month-old mice (Fig. S1a). As shown in Fig. 1e, a large number of cells showed signs of nuclear fragmentation, which is a characteristic of cellular necrosis. Quantitative analysis showed that *S. aureus*-infected femurs of 10-month-old mice had much larger necrotic area than that of 8-week-old mice (Fig. 1f). Consistent with this pathological change, immunostaining of phosphorylated mixed lineage domain-like (p-MLKL), the executor of necroptotic cell death, demonstrated a significantly increased expression in the bone marrow surrounding the infected implant in 10-month-old mice (Fig. 1g and h, Fig. S1b). These data indicate a compromised host defense against *S. aureus* at the early stage of infection in the bone marrow in middle-aged mice.

**Fig. 2:**
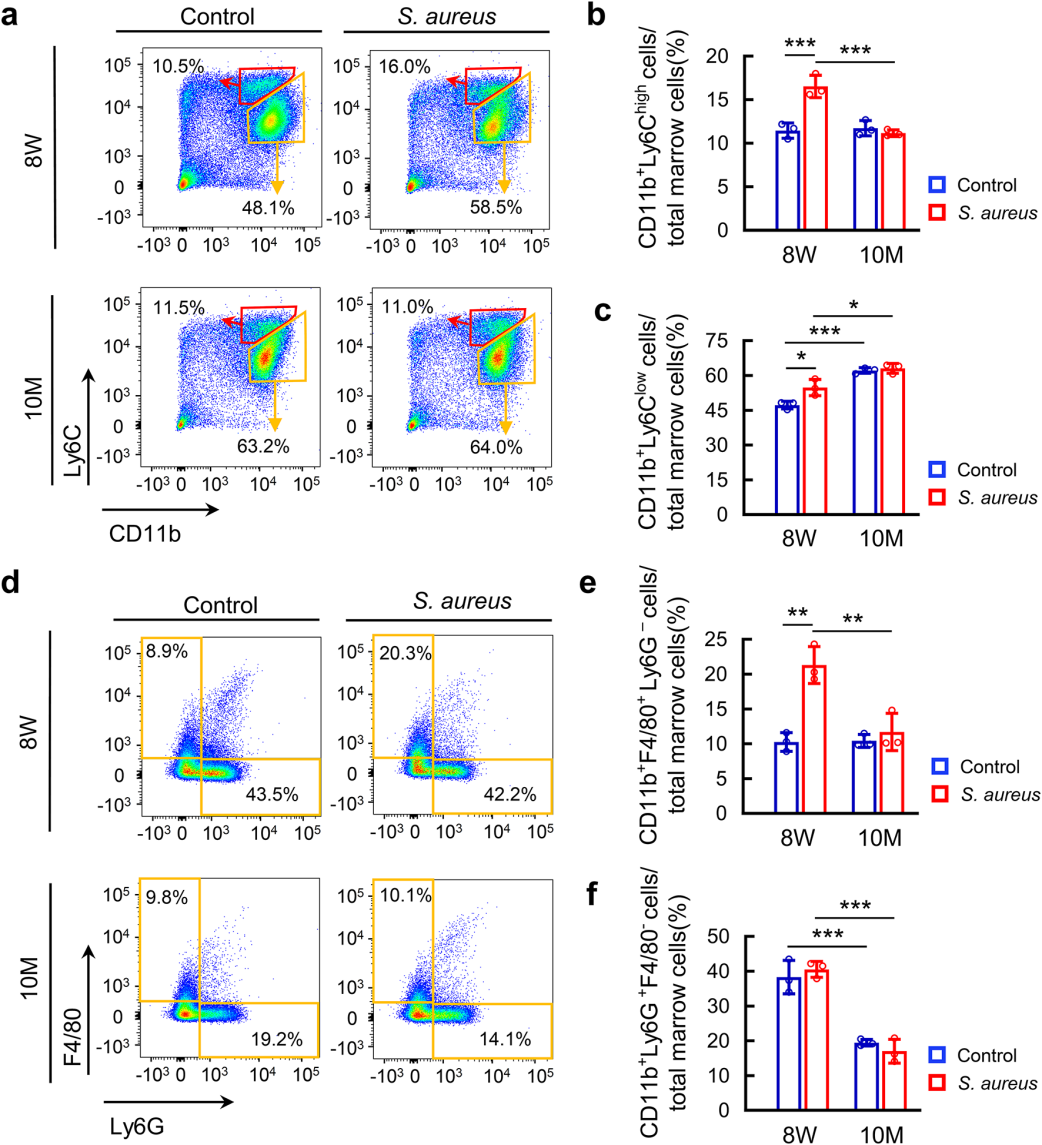
The recruitment of innate immune cells in *S. aureus*-infected femurs differs between 8-week-old mice and 10-month old mice. (**a**) Representative flow cytometry plots for CD11b^+^Ly6C^high^ inflammatory monocytes and CD11b^+^Ly6C^low^ non-inflammatory monocytes, and the percentages of CD11b^+^Ly6C^high^ inflammatory monocytes (**b**) and CD11b^+^Ly6C^low^ non-inflammatory monocytes (**c**) in total marrow cells. (**d**) Representative flow cytometry plots for CD11b^+^F4/80^+^Ly6G^−^ macrophages and CD11b^+^Ly6G^+^F4/80^−^ neutrophils, and the percentages of CD11b^+^F4/80^+^Ly6G^−^ macrophages (**e**) and CD11b^+^Ly6G^+^F4/80^−^ neutrophils (**f**) in the total marrow cells. *n* = 3/ group. One-way ANOVA with Bonferroni *post hoc* test, *p < 0.05, **p < 0.01, ***p < 0.001

To determine whether the above pathological changes were accompanied by alterations in innate immune response, we then used flow cytometry to quantify monocytes, macrophages and neutrophils in *S. aureus*-infected femurs in young and middle-aged mice. The gating strategy shown in Fig S2 was used to identify CD11b^+^Ly6C^high^ inflammatory monocytes, CD11b^+^Ly6C^low^ non-inflammatory monocytes, CD11b^+^F4/80^+^Ly6G^−^ macrophages and CD11b^+^Ly6G^+^F4/80^−^ neutrophils. We observed an increase in the proportion of Ly6C^high^ monocytes in the *S. aureus*-infected bone marrow of 8-week-old mice compared with control ones, but not in 10-month-old mice compared with its controls (Fig. 2a and b). Additionally, we detected a significant decrease in the proportion of Ly6C^high^ monocytes in infected femurs of 10-month-old mice compared with that of 8-week-old mice (Fig. 2a and b). Interestingly, there were no age-associated changes in the proportion of Ly6C^high^ monocytes between 10-month-old and 8-week-old mice under non-infection condition (Fig. 2a and b). Similarly, the proportion of CD11b^+^Ly6C^low^ cells increased in the bone marrow after 3 days of *S. aureus* infection in 8-week-old mice but not in 10-month-old mice (Fig. 2a and c). Notably, both non-infected and infected femurs of 10-month-old mice had a higher proportion of Ly6C^low^ cells compared with 8-week-old mice (Fig. 2a and c). Consistent with the changes in Ly6C^high^ inflammatory monocytes, we also observed an increased proportion of F4/80^+^ macrophages in *S. aureus*-infected femurs in 8-week-old mice compared with control ones and a decreased proportion of these cells in the bone marrow in 10-month-old mice (Fig. 2d and e). Interestingly, 3 days of *S. aureus* infection did not change the neutrophil population in the femoral bone marrow of either 8-week-old mice or 10-month-old mice (Fig. 2d and f). Unexpectedly, we found a striking decrease in the proportion of Ly6G^+^ neutrophils in the bone marrow of 10-month-old mice compared with 8-week-old mice under both infected and non-infected conditions (Fig. 2d and f). Besides, we didn’t find any changes in the percentage of CD3^+^CD4^+^ and CD3^+^CD8^+^ cell populations by day 3 post-infection (Fig. S3). Collectively, there were increased numbers of inflammatory monocytes and macrophages in *S. aureus*-infected femurs of young mice but not in middle-aged ones.

### Bone marrow monocytes in middle-aged mice have reduced expression of CXCL9/10 after *S. aureus* infection

**Fig. 3:**
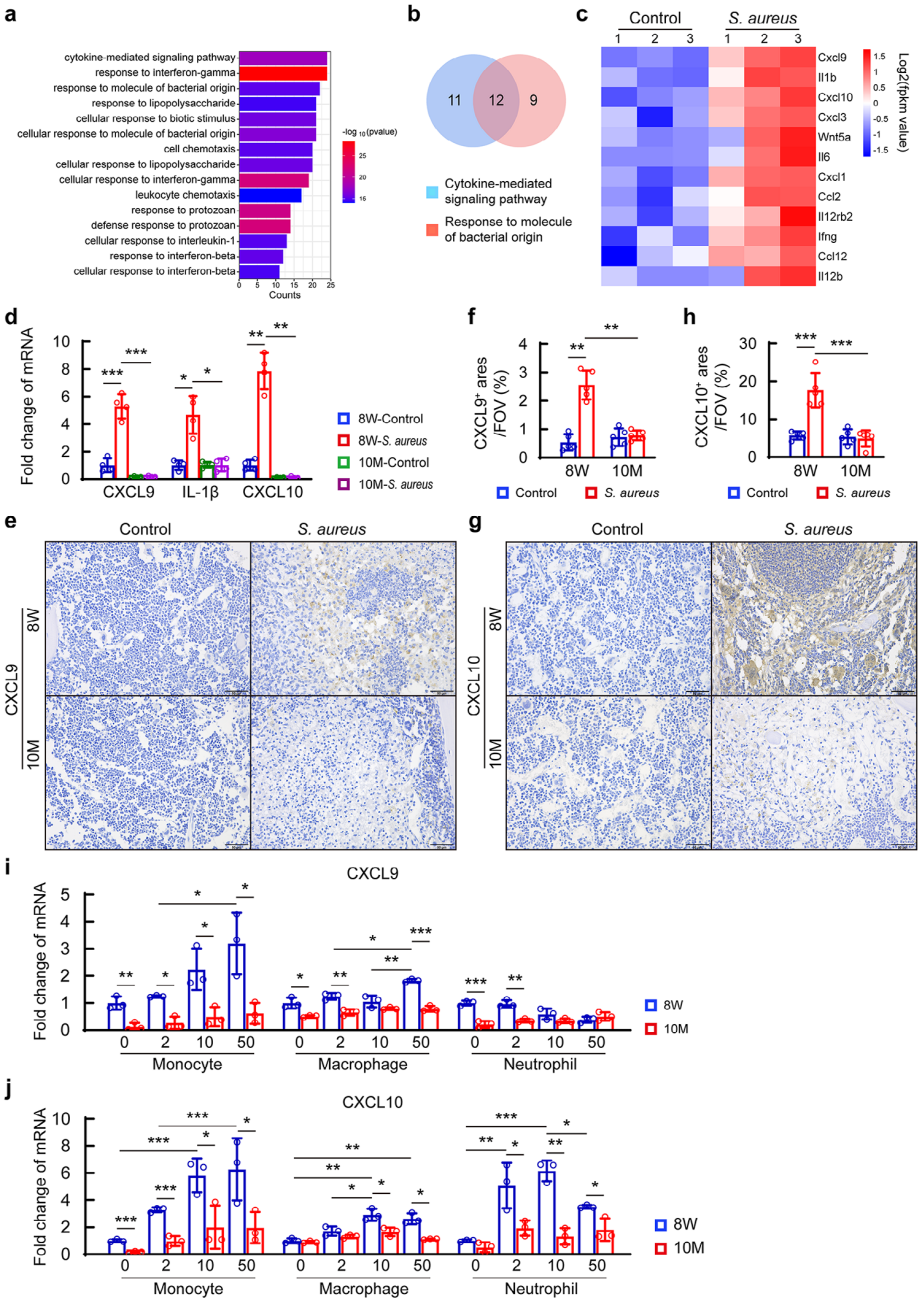
The expression of CXCL9 and CXCL10 are highly activated by *S. aureus* challenge in bone marrow monocytes of 8-week-old mice. (**a**) Gene ontology (GO) enrichment analysis of biological processes for upregulated differentially expressed genes (DEGs). The transcriptome data of *S. aureus*-infected bone and control ones in young mice on day 3 after surgery (GSE166522) were analyzed by bioinformatics. (**b**) Venn diagram of overlapping DEGs between cytokine-mediated signaling pathway and response to molecule of bacterial origin. (**c**) Heatmap of 12 overlapping DEGs. (**d**) mRNA expression of the top 3 highly up-regulated genes (CXCL9, IL-1β, CXCL10) in bone tissue on day 3 after surgery. *n* = 4/ group. One-way ANOVA with Dunnett’s T3 *post hoc* test, *p < 0.05, **p < 0.01, ***p < 0.001. (**e**) Representative images of immunohistochemical staining for CXCL9 in *S. aureus*-infected femurs and controls on day 3 after surgery, scale bar = 50 μm. (**f**) The percentages of CXCL9-positive stained area in the area of field of view (FOV) in bone marrow. *n* = 5/ group. One-way ANOVA with Dunnett’s T3 *post hoc* test, **p < 0.01. (**g**) Representative images of CXCL10 immunohistochemical staining in bone tissue on day 3 after surgery, scale bar = 50 μm. (**h**) The percentages of CXCL10-positive stained area in the area of FOV in bone marrow. *n* = 5/ group. One-way ANOVA with Bonferroni *post hoc* test, ***p < 0.001. mRNA expression of CXCL9 (**i**) and CXCL10 (**j**) in monocytes, macrophages and neutrophils isolated from bone marrow of 8-week-old mice and 10-month-old mice. Cells were treated with various MOI (0, 2, 10 and 50) of *S. aureus*, and total RNA were collected after 12 h. *n* = 3/group, One-way ANOVA with Bonferroni *post hoc* test, *p < 0.05, **p < 0.01, ***p < 0.001

The observations described above prompted us to explore the molecular process that might give rise to the alterations in innate immune response in a mouse model of *S. aureus* osteomyelitis. We analyzed the transcriptomic data of *S. aureus*-infected mice femurs and control ones by day 3 after infection (GEO: GSE166522) from our previous work [21]. Gene Ontology (GO) biological pathway (BP) enrichment analysis of the 96 upregulated differentially expressed genes (DEGs) revealed that 3 days of *S. aureus* infection in bone is associated with cytokine-mediated signaling pathway, response to interferon-gamma, cellular response to molecular bacterial origin, and other processes (Fig. 3a). Notably, the top 3 leading-edge genes of cytokine-mediated signaling pathway and response to molecule of bacterial origin processes contained CXCL9, IL-1β, and CXCL10 (a family member of CXCL9) (Fig. 3b and c). We further evaluated the mRNA expression of these 3 genes in the bone marrow in *S. aureus*-infected femurs from 8-week-old and 10-month-old mice. We observed significantly increased expression of CXCL9 and CXCL10 (CXCL9/10) and IL-1β in the bone marrow from *S. aureus*-infected femurs in 8-week-old mice (Fig. 3d). In addition, the basal levels of CXCL9/10 mRNA expression were considerably down-regulated in the bone marrow from 10-month-old mice compared with those from 8-week-old mice. Moreover, *S. aureus* infection failed to stimulate the expression of these 3 genes in the bone marrow from 10-month-old mice (Fig. 3d). Examinations of the protein levels of CXCL9/10 by immunohistochemistry confirmed upregulated expression of them in *S. aureus*-infected femurs from 8-week-old mice, and compromised activation of their expression in *S. aureus*-infected femurs from 10-month-old mice (Fig. 3e-h).

CXCL9/10 can be expressed and secreted by a variety of cells, including myeloid cells and T cells [24, 25]. As T-cell cytokines in *S. aureus*-infected bone exhibit a late activation pattern typically observed between 7 and 14 days post-infection [26, 27], we did not assess the expression of CXCL9/10 in T cells by day 3 post-infection in our study. To evaluate cellular sources of CXCL9/10 in bone marrow myeloid cells during *S. aureus* infection, we isolated monocytes, bone marrow-derived macrophages (BMDMs) and neutrophils, which are main components of *S. aureus* abscess [28], from the femoral bone marrow in 8-week-old mice and 10-month-old mice, and challenged them with various MOI of *S. aureus*. The purity of isolated monocytes, neutrophils, and BMDMs from bone marrow were determined by flow cytometry with surface markers before further experiments. There was an average purity of 88.7% for CD11b^+^Ly6C^+^ monocytes, 88.37% for CD11b^+^Ly6G^+^ neutrophils and 94.93% for CD11b^+^F4/80^+^ macrophages, respectively (Fig. S4a-S4f), confirming the reliability of our cell isolations. In response to *S. aureus* challenge, monocytes represented the major cell type that had strikingly increased mRNA expression of both CXCL9 and CXCL10 in response to *S. aureus* challenge, while expression of CXCL9/10 was only partially increased or unchanged in macrophages and neutrophils upon *S. aureus* challenge (Fig. 3i and j). Notably, *S. aureus* failed to activate the expression of CXCL9/10 in all three types of cells from 10-month-old mice (Fig. 3i and j), consistent with the reduced protein levels of CXCL9/10 in the *S. aureus*-infected femurs from 10-month-old mice. In sum, our data suggest that monocytes might be the major cellular sources of CXCL9/10 upon *S. aureus* infection in the bone marrow, and that the local CXCL9/10 production response might be reduced in monocytes from middle-aged mice after *S. aureus* infection.

### Monocyte-derived CXCL9/10 is critical for the bactericidal function of neutrophils and macrophages from bone marrow

**Fig. 4:**
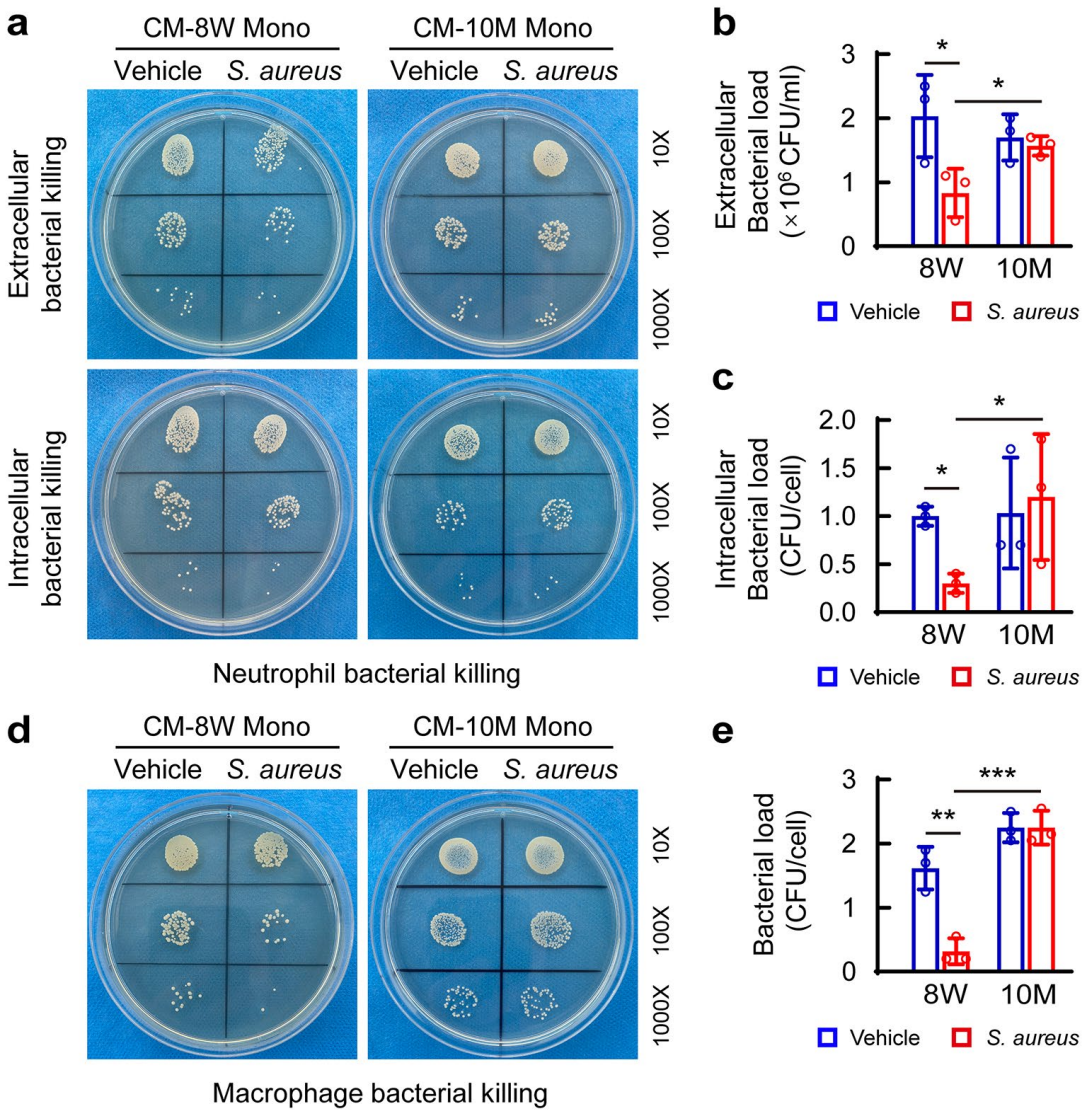
Conditioned medium of *S. aureus*-challenged monocytes from young mice improves the bactericidal function of neutrophils and macrophages. (**a**) Representative images of bacterial colonies on agar plates and quantification of extracellular bacterial burden in supernatants (**b**) and intracellular bacterial burden (**c**) of neutrophils. After neutrophils were pre-stimulated with conditioned medium of monocytes culture treated with PBS (CM Mono vehicle) or *S. aureus* (CM Mono *S. aureus*) for 4 h, *S. aureus* (MOI = 10) was co-cultured with pre-stimulated neutrophils for 30 min. CM-8 W Mono and CM-10 M Mono represent CM of monocytes isolated from the bone marrow of 8-week-old and 10-month-old mice, respectively. (**d**) Representative images of bacterial colonies on agar plates and (**e**) quantification of intracellular bacterial burden of BMDMs. After BMDMs were pre-stimulated with CM Mono vehicle or CM Mono *S. aureus* for 4 h, *S. aureus* (MOI = 10) was co-cultured with pre-stimulated BMDMs for 1 h. After removal of the extracellular *S. aureus*, the BMDMS were continued to be cultured with conditioned medium for 12 h. *n* = 3/ group. One-way ANOVA with Bonferroni *post hoc* test, *p < 0.05, **p < 0.01, ***p < 0.001

Recent studies have indicated critical roles of monocytes in immune escape, immune tolerance and wound repair during *S. aureus* infection [29–31]. Therefore, before evaluation of the function of CXCL9/10, we evaluated whether the effect of monocytes from young mice on innate immune responses might be different from that from middle-aged mice upon *S. aureus* infection. We isolated bone marrow monocytes from 8-week-old and 10-month-old mice, and challenged them with *S. aureus* or vehicle for 12 h, before we collected and filtered the conditioned medium (CM) for bacterial killing assay in neutrophils or macrophages. Flow cytometry analysis showed that predominant neutrophils isolated from Percoll were CMFDA^+^PI^−^ viable cells and 30 min of *S. aureus* challenge had no much effect on the percentage of CMFDA^+^PI^−^ neutrophils (Fig. S5a and S5b). In addition, there was a notable increase in DHE-labelled ROS levels compared with non-infected neutrophils (Fig. S5c and S5d), indicating reactiveness of the isolated neutrophils to *S. aureus* infection. We found that neutrophils treated with CM of *S. aureus*-challenged monocytes from 8-week-old mice (CM-8 W Mono-*S. aureus*) had significantly fewer extracellular and intracellular bacterial colonies compared with those treated with CM of vehicle-treated monocytes from 8-week-old mice (CM-8 W Mono-Vehicle) (Fig. 4a-c). Noticeably, neutrophils treated with CM of *S. aureus*-challenged monocytes from 10-month-old mice (CM-10 M Mono-*S. aureus*) had similar extracellular and intracellular bacterial loadings with those treated with CM of vehicle-treated monocytes from 10-month-old mice (CM-10 M Mono-Vehicle), and had a strikingly higher bacterial loading compared with those treated with CM-8 W Mono-*S. aureus* (Fig. 4a-c). Similar to the observations in neutrophils, a significant decreased bacterial loading was observed in macrophages treated with CM-8 W Mono-*S. aureus* compared with those treated with CM-8 W Mono-Vehicle. Meanwhile, CM-10 M Mono-*S. aureus*-treated bone marrow-derived macrophages (BMDMs) had a similar bacterial loading with CM-10 M Mono-Vehicle-treated ones, but had a strikingly higher bacterial expansion compared with CM-8 W Mono-*S. aureus*-treated ones (Fig. 4d and e). These results suggest that *S. aureus*-challenged monocytes from young mice may improve the bactericidal function of neutrophils and macrophages, but those from middle-aged mice may not.

**Fig. 5:**
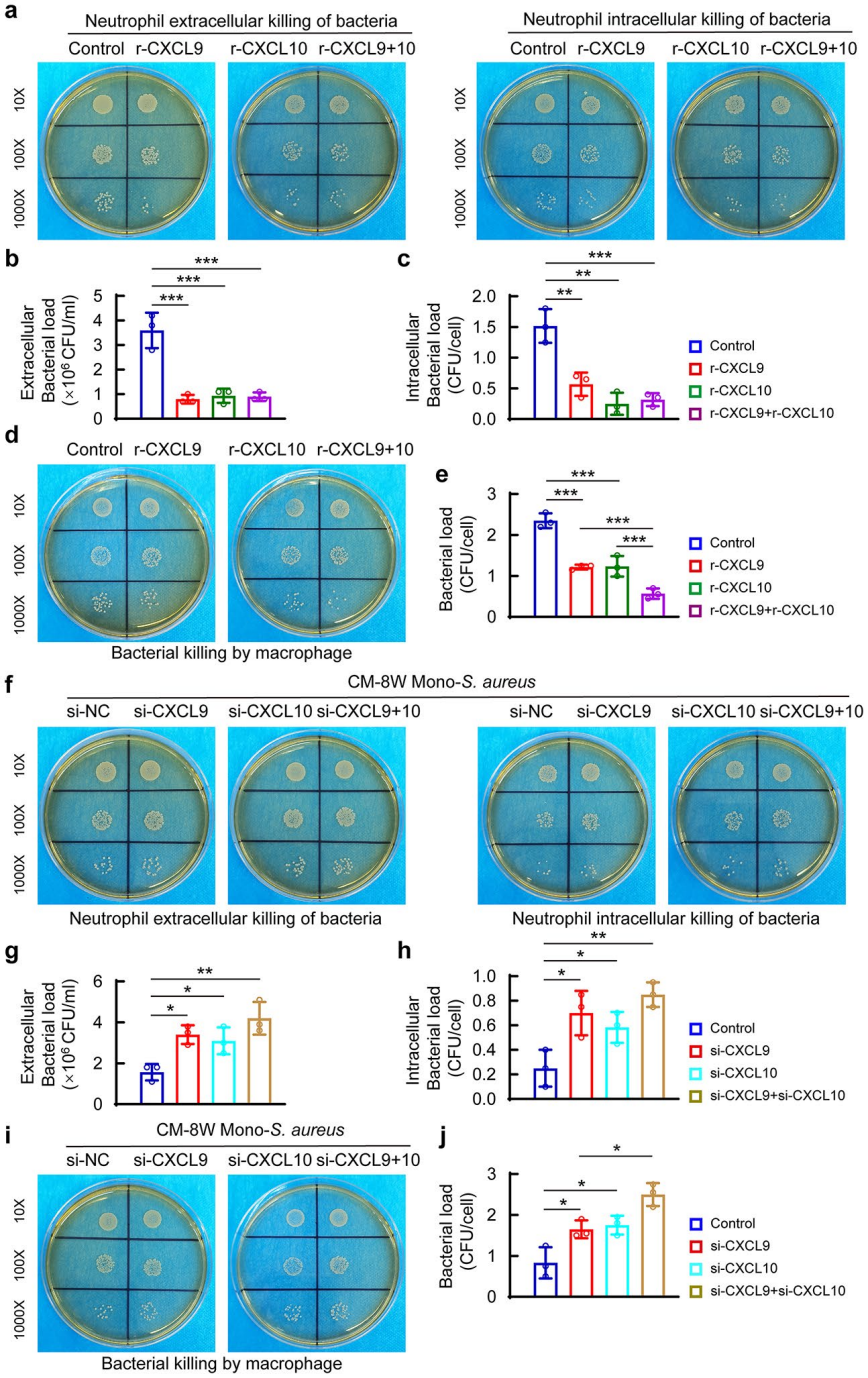
Monocyte-derived CXCL9/10 evoked by *S. aureus* enhance the bactericidal function of neutrophils and macrophages. (**a**) Representative images of the remaining extracellular and intracellular *S. aureus* colonies inoculated in TSA plates, and quantitative analysis of extracellular (**b**) and intracellular (**c**) bacterial colonies of neutrophils culture. After neutrophils were pre-stimulated with 100 ng/ml of recombinant CXCL9 (r-CXCL9), recombinant CXCL10 (r-CXCL10), or a combination of them for 4 h, cells were challenged with *S. aureus* at MOI of 10 for 30 min. *n* = 3/ group. One-way ANOVA with Bonferroni *post hoc* test, **p < 0.01, ***p < 0.001. (**d**) Representative images of intracellular *S. aureus* of BMDMs inoculated in TSA and (**e**) quantification of bacteria colonies of BMDMs pre-treated with r-CXCL9, r-CXCL10, and a combination of them. *n* = 3/ group. One-way ANOVA with Bonferroni *post hoc* test, ***p < 0.001. (**f**) Representative images of the remaining extracellular and intracellular *S. aureus* colonies inoculated in TSA plates from neutrophils culture, and quantitative analysis of extracellular (**g**) and intracellular (**h**) bacterial colonies. After neutrophils were pre-stimulated for 4 h with CM-8 W Mono *S. aureus* that had been treated with recombinant siRNA for CXCL9 (si-CXCL9), CXCL10 (si-CXCL10), a combination of si-CXCL9 and si-CXCL10, or negative control (si-NC), cells were then challenged with *S. aureus* at MOI of 10 for 30 min. CM-8 W Mono-*S. aureus* represents the CM of monocytes isolated from the bone marrow of 8-week-old and challenged by *S. aureus*. *n* = 3/ group. One-way ANOVA with Bonferroni *post hoc* test, *p < 0.05, **p < 0.01. (**i**) Representative images of intracellular *S. aureus* colonies of BMDMs and (**j**) quantification of bacterial colonies of BMDMs pretreated with CM-8 W Mono-*S. aureus* with the knockdown of CXCL9 and CXCL10. *n* = 3/ group. One-way ANOVA with Bonferroni *post hoc* test, *p < 0.05

The chemotaxis function of CXCL9/10 is known as its action on its cognate receptor C-X-C motif chemokine receptor 3 (CXCR3) in immune cells such as macrophages, neutrophils and other immune cells [32]. Moreover, recent literature has demonstrated the protective role of CXCR3 blockade against virus infection [33, 34]. These prompted us to determine whether the presence of CXCL9/10 upon *S. aureus* infection might be functionally relevant to the bactericidal function of innate immune cells. We pretreated neutrophils in vitro with recombinant mouse CXCL9, CXCL10, and a combination of them to measure the bacterial killing capacity of neutrophils. Interestingly, we found that the number of extracellular bacterial colonies was significantly decreased in the wells treated by CXCL9 or CXCL10 (Fig. 5a and b). Meanwhile, we also observed a significant decline in intracellular bacterial colonies in the neutrophils treated by CXCL9 or CXCL10 (Fig. 5a and c). Whereas, the combination of CXCL9 and CXCL10 treatment didn’t further reduce either extracellular or intracellular bacterial load of neutrophils compared with treatment with either CXCL9 or CXCL10 (Fig. 5a-c). In addition, there was a significant decrease in the number of bacterial colonies in the BMDMs treated by either CXCL9 or CXCL10, and a combination of CXCL9 and CXCL10 further reduced bacterial load in BMDMs (Fig. 5d and e).

We next investigated whether the elevated expression of CXCL9/10 in monocytes in response to *S. aureus* infection might be responsible for the elevated bactericidal activity of neutrophils and macrophages. We isolated primary monocytes from 8-week-old mice bone marrow, and treated them with siRNA fragments targeting CXCL9 or CXCL10 or control ones for 48 h before *S. aureus* infection. The knock-down efficiency of siRNA fragments was evaluated by qPCR, and si-CXCL9-3 and si-CXCL10-2 fragments were chosen for further experiments (Fig. S6). CM-8 W Mono-*S. aureus* with knockdown of CXCL9, CXCL10, or both of them was collected after 12 h of infection before treatment with neutrophils or macrophages. We found that CM-8 W Mono-*S. aureus* with knockdown of either CXCL9 or CXCL10 significantly increased the numbers of both extracellular and intracellular bacterial colonies of *S. aureus*-infected neutrophils (Fig. 5f-h). However, knockdown of both CXCL9 and CXCL10 didn’t further increase the bacterial load in neutrophils compared with knockdown of either CXCL9 or CXCL10 alone (Fig. 5f-h). In addition, the number of bacterial colonies in BMDMs was also considerably increased by the treatment of CM-8 W Mono-*S. aureus* with knockdown of CXCL9 or CXCL10 (Fig. 5i and j). Interestingly, knockdown of both CXCL9 and CXCL10 further increased the bacterial load in BMDMs compared with knockdown of either CXCL9 alone (Fig. 5i and j). The above data indicate that CXCL9/10 production from monocytes activated by *S. aureus* infection may enhance the bactericidal function of neutrophils and macrophages.

### CXCR3 signaling in neutrophils and macrophages promotes their bactericidal function

**Fig. 6:**
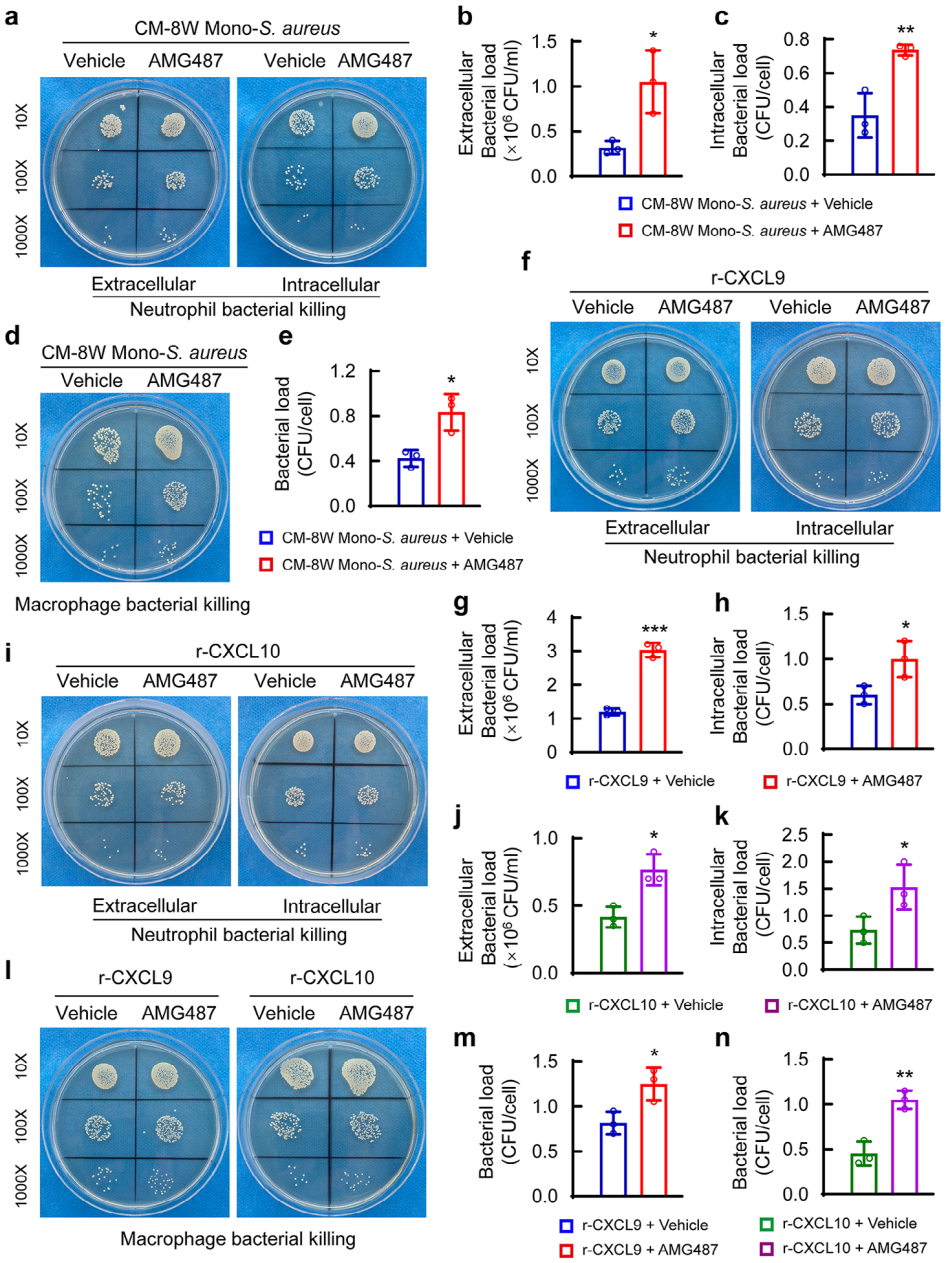
CXCR3 signaling in neutrophils and macrophages promotes their bactericidal function. (**a**) Representative images of the remaining extracellular and intracellular *S. aureus* colonies of neutrophils inoculated in TSA, and quantitative analysis of extracellular (**b**) and intracellular (**c**) bacterial colonies. Neutrophils were pre-stimulated with AMG487 (500 µM), an inhibitor of CXCR3, for 1 h, and then cultured in CM-8 W Mono-*S. aureus* for 4 h. Next, cells were challenged with *S. aureus* (MOI = 10) for 30 min, and bactericidal function were evaluated. *n* = 3/ group. Student’s *t* test, *p < 0.05, **p < 0.01. (**d**) Representative images of the remaining intracellular *S. aureus* colonies of BMDMs inoculated in TSA, and (**e**) quantification of bacterial colonies. BMDMs were pre-stimulated with 500 µM AMG487 for 1 h, and then further stimulated with CM-8 W Mono-*S. aureus* for 4 h. Next, cells were challenged with *S. aureus* (MOI = 10) for 1 h. After removal of the extracellular *S. aureus*, culture of the BMDMS was continued with CM-8 W Mono-*S. aureus* and AMG487 for 12 h and bacterial loading were evaluated. *n* = 3/ group. Student’s *t* test, *p < 0.05. (**f** and **i**) Representative images of the remaining extracellular and intracellular *S. aureus* colonies of neutrophils inoculated in TSA, and quantitative analysis of extracellular (**g** and **j**) and intracellular (**h** and **k**) bacterial colonies. Neutrophils were pre-stimulated with 500 µM AMG487 for 1 h, and then further treated with 100 ng/ml of r-CXCL9 or r-CXCL10 for 4 h. Next, *S. aureus* (MOI = 10) was co-cultured with pre-stimulated neutrophils for 30 min. Finally, the extracellular and intracellular bacterial loading were evaluated. *n* = 3/ group. Student’s *t* test, *p < 0.05, ***p < 0.001. (**l**) Representative images of remaining intracellular bacteria of BMDMs inoculated on TSA and (m and n) quantification of *bacterial* colonies in BMDMs. BMDMs were pre-stimulated with 500 µM AMG487 for 1 h, and then further stimulated with 100 ng/ml of r-CXCL9 or r-CXCL10 for 4 h. Next, cells were challenged with *S. aureus* (MOI = 10) for 1 h. After removal of the extracellular *S. aureus*, culture of the BMDMS was continued with AMG487 and with the presence or absence of r-CXCL9 or r-CXCL10 for 12 h. *n* = 3/ group. Student’s *t* test, *p < 0.05, **p < 0.01

To confirm the role of monocytes-originated CXCL9/10 in the activities of neutrophils and macrophages, we then determined whether blocking CXCR3 signaling could lead to an alteration in their bactericidal function. Blocking CXCR3 with AMG487 increased both the extracellular and intracellular bacterial burdens of neutrophils (Fig. 6a-c). A similar phenotype with an increased *S. aureus* burden was observed in AMG487-treated BMDMs (Fig. 6d and e). A recent study indicated a bactericide/bacteriostatic function of Teleost CXCL10 against fish pathogens [35]. Thus, to exclude the possible direct effect of CXCL9/10 on suppressing *S. aureus*, we explored whether inhibition of CXCR3 might block the effect of CXCL9/10 on the bactericidal function of neutrophils and macrophages. As expected, AMG487 treatment significantly ablated the positive effect of CXCL9 or CXCL10 on extracellular and intracellular bacterial killing of neutrophils (Fig. 6f-k). In addition, either CXCL9- or CXCL10-driven antimicrobial activity of macrophages against *S. aureus* was also blocked by AMG487 (Fig. 6l-n). The above data demonstrated that *S. aureus*-challenged monocytes from young mice may enhance bactericidal activity of neutrophils and macrophages through the CXCL9/10-CXCR3 axis.

### Reduced production of CXCL9/10 in monocytes upon *S. aureus* challenge contributes to compromised antimicrobial function in middle-aged mice

**Fig. 7:**
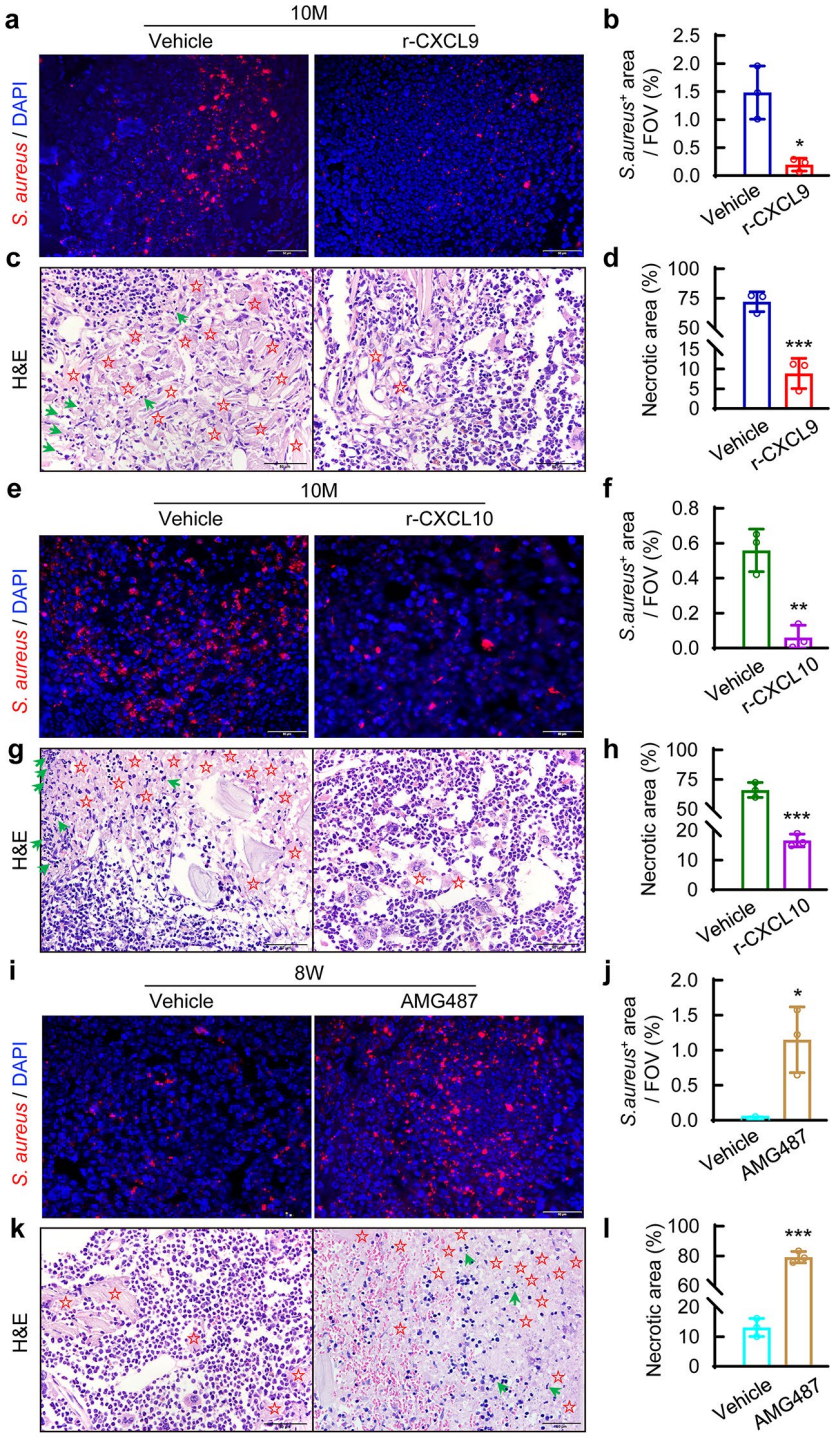
CXCL9/CXCL10-CXCR3 signaling mediates age-related lesions in the acute phase of *S. aureus* osteomyelitis in mice. (**a**) Representative images of immunofluorescence staining for *S. aureus* and (**b**) quantification of *S. aureus*-positive stained area per area of field of view (FOV). (**c**) Representative images of H&E staining and (**d**) quantification of necrotic area per area of FOV. 10-month-old mice were injected with 1 µl r-CXCL9 (100 ng/µl) into the bone marrow where the implant was placed and *S. aureus* was injected, and right femurs were collected by day 3 after surgery for further analysis. *n* = 3/group. Scale bar = 50 μm. (**e**) Representative images of immunofluorescence staining for *S. aureus* and (**f**) quantification of *S. aureus*-positive stained area per FOV area. (**g**) Representative images of H&E staining and (**h**) quantification of necrotic area per area of FOV. 10-month-old mice were injected with 1 µl r-CXCL10 (100 ng/µl) into the bone marrow where the implant was placed and *S. aureus* was injected, and right femurs were collected by day 3 after surgery for further analysis. *n* = 3/group. Scale bar = 50 μm. (**i**) Representative images of immunofluorescence staining for *S. aureus* and (**j**) quantification of *S. aureus*-positive stained area per FOV area. (**k**) Representative images of H&E staining and (**l**) quantification of necrotic area per area of FOV. After being implanted and infected with *S. aureus* or treated with vehicle in right femurs, 8-week-old mice were injected subcutaneously with AMG487 (5 mg/kg, twice a day), right femurs were collected for further analysis by day 3 after surgery. *n* = 3/group, Student’s *t* test. *p < 0.05, **p < 0.01, ***p < 0.001. Red fluorescence indicates *S. aureus* positive staining, and blue DAPI stained nucleus. The red star indicates the cellular lytic changes, the green arrow nuclear fragmentation

To test the protective role of CXCL9/10 in an early stage of *S. aureus* osteomyelitis in mice, we administrated recombinant CXCL9 or CXCL10 to 10-month-old mice with *S. aureus* osteomyelitis. We found that either CXCL9- or CXCL10-treated mice had a strikingly smaller bacterial burden in bone marrow compared with vehicle-treated ones, as revealed by immunofluorescence staining of *S. aureus* in infected femurs (Fig. 7a, b, e and f). Additionally, vehicle-treated mice showed extensive cell necrosis and bone marrow lesions around the *S. aureus*-infected site, while either CXCL9 or CXCL10 treatment significantly improved cellular morphology and tissue structure in *S. aureus*-infected bone marrow (Fig. 7c, d, g and h).

To determine whether enhanced production of CXCL9/10 in monocytes from young mice might account for increased bactericidal function in neutrophils and macrophages, we applied AMG487 to 8-week-old mice with *S. aureus* osteomyelitis. Immunofluorescence staining for *S. aureus* showed significant expansion of bacteria in the bone marrow in AMG487-treated mice compared with vehicle-treated ones (Fig. 7i and j). In line with the increased bacterial loading in bone marrow, cells necrosis and bone marrow lesions were considerably increased after AMG487 treatment in 8-week-old mice (Fig. 7k and l).

## Discussion

There are major gaps in understanding the cellular and molecular mechanisms underlying the age-associated decline in the resistance to *S. aureus* infection in skeleton. Here, using a mice model of *S. aureus* osteomyelitis, we have identified bone marrow monocytes as a predominant cellular source of CXCL9/10 upon *S. aureus* challenge. Moreover, we provide a unique insight into the CXCL9 and CXCL10, known to have a strong effect on recruiting other immune cells by acting on the CXCR3 receptor. We have found that they may promote bactericidal function of macrophages and neutrophils in bone marrow. Finally, we have provided evidence that declined production of CXCL9/10 in monocytes upon *S. aureus* challenge may be responsible for the reduced antibacterial activity of innate immune cells in middle-aged mice. All the data gained by this study may facilitate development of feasible therapeutic approaches for *S. aureus* osteomyelitis in aged hosts.

An interesting finding of this study is that we have identified a novel function of monocytes as a critical regulator of the innate immunity against *S. aureus* which may decline with aging. One of known and primary functions of monocytes is differentiation into macrophages and dendritic cells to control infection [36–38]. Our work has extended this observation to show that monocytes are critical in control of the antimicrobial defense function of macrophages and neutrophils upon *S. aureus* infection. Mounting evidence indicates a diminished response in monocytes from whole blood of middle-aged or aged individuals challenged with pattern recognition receptor agonists, resulting in a substantially reduced NF-κB signaling and decreased production of proinflammatory cytokines such as IFN-α, IFN-γ, IL-1β [39, 40]. Our findings also suggest a diminished response in bone marrow monocytes of middle-aged mice following *S. aureus* infection, leading to impaired bactericidal function of macrophages and neutrophils and increased bacterial burden. These findings suggest that the decreased responsiveness and altered phenotype of monocytes may contribute to age-related immune system impairment, ultimately rendering elderly individuals more susceptible to and experiencing more severe infections [41–43]. Consequently, targeting monocytes with specific therapies could be a promising approach for combating *S. aureus* osteomyelitis in elderly population.

The most important finding of the present study is that CXCL9/10 may play a critical role in controlling the bacterial killing activities of macrophages and neutrophils during *S. aureus* infection. CXCL9/10, known to be interferon (IFN)-induced angiostatic CXC chemokines, can be expressed by a variety of cells, such as T cells, monocytes, neutrophils, endothelial cells and stromal cells [32, 34, 44]. Our previous work identified an up-regulated expression of CXCL9/10 in young mice femurs with *S. aureus* osteomyelitis [21]. In the present work, we further revealed that CXCL9/10 production was predominantly induced in monocytes upon *S. aureus* infection, and its levels in both basal and *S. aureus*-challenged conditions were declined in middle-aged mice. Although accumulating evidence have supported the redundant functions of CXCL9 and CXCL10 in controlling recruitment, differentiation and proliferation of immune cells in various disease, such as tumor development and viral infections [34, 44, 45], their activities in *S. aureus* infection remain unclear. Our findings here advanced current understanding of CXCL9/10 function in host defense by connecting monocytes activities with bactericidal functions of macrophages and neutrophils. Furthermore, our findings indicate highly activated CXCL10 in neutrophils challenged with *S. aureus* challenge in vitro, consistent with previous studies observing elevated CXCL10 and CXCR3 in neutrophils during infection with *Salmonella*, *Aspergillus*, or respiratory virus [46–48]. Thus, the less responsiveness of neutrophils to combination treatment with CXCL9 and CXCL10 proteins or their knockdown in monocytes may be attributed to heightened activation of CXCL10 expression in neutrophils during *S. aureus* challenge, or due to differential interaction between CXCL9 and CXCL10 in immune cells [44]. Additional investigations are needed to delineate the cellular sources of CXCL9/10 and their altered response to *S. aureus* infection during aging in vivo.

The translocation of phosphorylated MLKL to the cell membrane is a hallmark of necroptosis [49], a process that plays an important role in mounting antimicrobial response. Activation of MLKL signaling in neutrophils may promote the production of ROS and the extrusion of bacteriostatic NETs [50]. In models of skin infection or sepsis, MLKL knock-out mice exhibited high bacterial loads [51], underscoring the importance of MLKL in host defense. Thus, the increased levels of phosphorylated MLKL observed in both 8-week-old and 10-month-old mice indicate a host defense mechanism against *S. aureus* infection. However, the activation of MLKL in macrophages could also lead to membrane permeabilization and the release of pro-inflammatory cytokines such as IL-1β and CXCL10 [52, 53], which have been associated with inflammation-induced tissue damage [53, 54]. Interestingly, our study revealed increased levels of phosphorylated MLKL but a decrease in CXCL10 production in 10-month-old mice following *S. aureus* infection, potentially due to impaired migration of immune cells like monocytes and neutrophils observed in the present study.

Our previous research, along with other studies, has extensively documented the progressive histopathological changes occurring in *S. aureus*-infected bone among young mice aged between 8 and 10 weeks. By day 14 post-infection, evidence of periosteal bone formation and bone marrow abscess formation becomes apparent, followed by the emergence of large abscesses, sequestra, intense marrow fibrosis, and femur deformities by day 28 [21, 55, 56]. While our investigations did not extend to evaluating long-term pathological changes in *S. aureus*-infected bone in aging mice, the observation of cell necrosis in the bone marrow of middle-aged mice, even during the acute stage of infection, suggests a potentially more severe bone destruction process in aged mice. This has significant clinical implications, as older patients with osteomyelitis often experience poor outcomes, including heightened rates of fragility fractures and increased long-term mortality risk [6, 7]. Further research is needed to unravel the mechanisms underlying cellular necrosis during the acute stage of *S. aureus* osteomyelitis in aged bone marrow.

In conclusion, this study has uncovered that monocyte-derived CXCL9/10 augments the antibacterial activities of macrophages and neutrophils upon *S. aureus* challenge, and an age-dependent declined production of CXCL9/10 in monocytes upon *S. aureus* infection contributes to reduced antimicrobial defense in skeleton. This work reveals a novel function of CXCL9/10-CXCR3 axis as a vital component of innate immunity against *S. aureus* invasion, suggesting that promoted production of CXCL9/10 in monocytes might be an effective strategy in the combat against *S. aureus* infection in aged skeleton.

### Electronic supplementary material

Below is the link to the electronic supplementary material.

Supplementary Material 1

## Data Availability

All data generated or analyzed during this study are included in this published article and its supplementary information files.

## Abbreviations

S. aureus: Staphylococcus aureus
CXCL9: C-X-C motif chemokine ligand 9
CXCL10: C-X-C motif chemokine ligand 10
CXCR3: C-X-C motif chemokine receptor 3
TSB: Tryptic soy broth
OD: Optical density
CFU: Colony forming unit
PBS: Phosphate buffer saline
EDTA: Ethylenediaminetetraacetic acid
H&E: Hematoxylin and eosin
MLKL: Mixed-lineage kinase domain-like
DAB: 3,3-N-Diaminobenzidine tertrahydrochloride
DEGs: Differential expressed genes
GO: Gene Ontology
BMDMs: Bone marrow derived macrophages
ROS: Reactive oxygen species
NETs: Neutrophil extracellular traps
DHE: Dihydroethidium
CM: Conditional medium
MOI: Multiplicity of infection
qPCR: Quantitative real-time PCR

## References

[1] Ren Y, Liu L, Sun D, Zhang Z, Li M, Lan X, Ni J, Yan MM, Huang W, Liu ZM, Peng A, Zhang Y, Jiang N, Song K, Huang Z, Bi Q, Zhang J, Yang Q, Yang J, Liu Y, Fu W, Tian X, Wang Y, Zhong W, Song X, Abudurexiti A, Xia Z, Jiang Q, Shi H, Liu X, Wang G, Hu Y, Zhang Y, Yin G, Fan J, Feng S, Zhou X, Li Z, He W, Weeks J, Schwarz EM, Kates SL, Huang L, Chai Y, Bin Yu MD, Xie Z, Deng Z, Xie C (2023). Epidemiological updates of post-traumatic related limb osteomyelitis in China: a 10 years multicentre cohort study. Int J Surg.

[2] Jung N, Ernst A, Joost I, Yagdiran A, Peyerl-Hoffmann G, Grau S, Breuninger M, Hellmich M, Kubosch DC, Klingler JH, Seifert H, Kern WV, Kaasch AJ, Rieg S (2021). Vertebral osteomyelitis in patients with *Staphylococcus aureus* bloodstream infection: evaluation of risk factors for treatment failure. J Infect.

[3] Conan Y, Laurent E, Belin Y, Lacasse M, Amelot A, Mulleman D, Rosset P, Bernard L, Grammatico-Guillon L (2021). Large increase of vertebral osteomyelitis in France: a 2010–2019 cross-sectional study. Epidemiol Infect.

[4] Walter N, Baertl S, Alt V, Rupp M (2021). What is the burden of osteomyelitis in Germany? An analysis of inpatient data from 2008 through 2018. BMC Infect Dis.

[5] Murillo O, Grau I, Lora-Tamayo J, Gomez-Junyent J, Ribera A, Tubau F, Ariza J, Pallares R (2015). The changing epidemiology of bacteraemic osteoarticular infections in the early 21st century. Clin Microbiol Infect.

[6] Hsieh E, Shiau S, Nolan O, Gibert CL, Bedimo RJ, Rodriguez-Barradas MC, Justice AC, Womack JA, Yin MT (2019). Increased fragility fracture rates in older men with osteomyelitis. Clin Infect Dis.

[7] Huang CC, Tsai KT, Weng SF, Lin HJ, Huang HS, Wang JJ, Guo HR, Hsu CC (2016). Chronic osteomyelitis increases long-term mortality risk in the elderly: a nationwide population-based cohort study. BMC Geriatr.

[8] Nikolich-Žugich J (2018). The twilight of immunity: emerging concepts in aging of the immune system. Nat Immunol.

[9] Panda A, Arjona A, Sapey E, Bai F, Fikrig E, Montgomery RR, Lord JM, Shaw AC (2009). Human innate immunosenescence: causes and consequences for immunity in old age. Trends Immunol.

[10] Silva MT (2010). When two is better than one: macrophages and neutrophils work in concert in innate immunity as complementary and cooperative partners of a myeloid phagocyte system. J Leukoc Biol.

[11] Sapey E, Greenwood H, Walton G, Mann E, Love A, Aaronson N, Insall RH, Stockley RA, Lord JM (2014). Phosphoinositide 3-kinase inhibition restores neutrophil accuracy in the elderly: toward targeted treatments for immunosenescence. Blood.

[12] Barkaway A, Rolas L, Joulia R, Bodkin J, Lenn T, Owen-Woods C, Reglero-Real N, Stein M, Vázquez-Martínez L, Girbl T, Poston RN, Golding M, Saleeb RS, Thiriot A, von Andrian UH, Duchene J, Voisin MB, Bishop CL, Voehringer D, Roers A, Rot A, Lämmermann T, Nourshargh S (2021). Age-related changes in the local milieu of inflamed tissues cause aberrant neutrophil trafficking and subsequent remote organ damage. Immunity.

[13] Inomata M, Xu S, Chandra P, Meydani SN, Takemura G, Philips JA, Leong JM (2020). Macrophage LC3-associated phagocytosis is an immune defense against *Streptococcus pneumoniae* that diminishes with host aging. Proc Natl Acad Sci USA.

[14] Blacher E, Tsai C, Litichevskiy L, Shipony Z, Iweka CA, Schneider KM, Chuluun B, Heller HC, Menon V, Thaiss CA, Andreasson KI (2022). Aging disrupts circadian gene regulation and function in macrophages. Nat Immunol.

[15] Kovacs EJ, Palmer JL, Fortin CF, Fülöp T, Goldstein DR, Linton PJ (2009). Aging and innate immunity in the mouse: impact of intrinsic and extrinsic factors. Trends Immunol.

[16] Frisch BJ, Hoffman CM, Latchney SE, LaMere MW, Myers J, Ashton J, Li AJ, Saunders J 2nd, Palis J, Perkins AS, McCabe A, Smith JN, McGrath KE, Rivera-Escalera F, McDavid A, Liesveld JL, Korshunov VA, Elliott MR, MacNamara KC, Becker MW, Calvi LM (2019) Aged marrow macrophages expand platelet-biased hematopoietic stem cells via Interleukin1B. JCI Insight 5(10):e124213 10.1172/jci.insight.124213 PMC6542605 30998506

[17] Lu Y, Luo Y, Zhang Q, Chen W, Zhang N, Wang L, Zhang Y (2023). Decoding the immune landscape following hip fracture in elderly patients: unveiling temporal dynamics through single-cell RNA sequencing. Immun Ageing.

[18] Barman PK, Shin JE, Lewis SA, Kang S, Wu D, Wang Y, Yang X, Nagarkatti PS, Nagarkatti M, Messaoudi I, Benayoun BA, Goodridge HS (2022). Production of MHCII-expressing classical monocytes increases during aging in mice and humans. Aging Cell.

[19] Wang C, Cheng Y, Li B, Qiu X, Hu H, Zhang X, Lu Z, Zheng F (2023). Transcriptional characteristics and functional validation of three monocyte subsets during aging. Immun Ageing.

[20] Liu Y, Xiao J, Cai J, Li R, Sui X, Zhang J, Lu T, Chen H, Chen G, Li H, Jiang C, Zhao X, Xiao C, Lei Y, Yao J, Lv G, Liang J, Zhang Y, Yang JR, Zheng J, Yang Y (2024). Single-cell immune profiling of mouse liver aging reveals Cxcl2 + macrophages recruit neutrophils to aggravate liver injury. Hepatology.

[21] Lin Y, Su J, Wang Y, Xu D, Zhang X, Yu B (2021). mRNA transcriptome analysis of bone in a mouse model of implant-associated *Staphylococcus aureus* osteomyelitis. Infect Immun.

[22] Wang X, Spandidos A, Wang H, Seed B (2012). PrimerBank: a PCR primer database for quantitative gene expression analysis, 2012 update. Nucleic Acids Res.

[23] Hidalgo A, Chilvers ER, Summers C, Koenderman L (2019). The neutrophil life cycle. Trends Immunol.

[24] Schädel P, Czapka A, Gebert N, Jacobsen ID, Ori A, Werz O (2023). Metabololipidomic and proteomic profiling reveals aberrant macrophage activation and interrelated immunomodulatory mediator release during aging. Aging Cell.

[25] Lu G, Zhang R, Geng S, Peng L, Jayaraman P, Chen C, Xu F, Yang J, Li Q, Zheng H, Shen K, Wang J, Liu X, Wang W, Zheng Z, Qi CF, Si C, He JC, Liu K, Lira SA, Sikora AG, Li L, Xiong H (2015). Myeloid cell-derived inducible nitric oxide synthase suppresses M1 macrophage polarization. Nat Commun.

[26] Yoon KS, Fitzgerald RH, Sud S, Song Z, Wooley PH (1999). Experimental acute hematogenous osteomyelitis in mice. II. Influence of *Staphylococcus aureus* infection on T-cell immunity. J Orthop Res.

[27] Rochford ETJ, Sabaté Brescó M, Zeiter S, Kluge K, Poulsson A, Ziegler M, Richards RG, O’Mahony L, Moriarty TF (2016). Monitoring immune responses in a mouse model of fracture fixation with and without *Staphylococcus aureus* osteomyelitis. Bone.

[28] Kobayashi SD, Malachowa N, DeLeo FR (2015). Pathogenesis of *Staphylococcus aureus* abscesses. Am J Pathol.

[29] Musilova J, Mulcahy ME, Kuijk MM, McLoughlin RM, Bowie AG (2019). Toll-like receptor 2-dependent endosomal signaling by *Staphylococcus aureus* in monocytes induces type I interferon and promotes intracellular survival. J Biol Chem.

[30] Müller MM, Baldauf C, Hornischer S, Klassert TE, Schneegans A, Behnert A, Pletz MW, Hagel S, Slevogt H (2023). *Staphylococcus aureus* induces tolerance in human monocytes accompanied with expression changes of cell surface markers. Front Immunol.

[31] Kratofil RM, Shim HB, Shim R, Lee WY, Labit E, Sinha S, Keenan CM, Surewaard BGJ, Noh JY, Sun Y, Sharkey KA, Mack M, Biernaskie J, Deniset JF, Kubes P (2022). A monocyte-leptin-angiogenesis pathway critical for repair post-infection. Nature.

[32] Van Raemdonck K, Van den Steen PE, Liekens S, Van Damme J, Struyf S (2015). CXCR3 ligands in disease and therapy. Cytokine Growth Factor Rev.

[33] Singh A, Ghosh R, Guchhait P (2023). CXCR3 antagonist rescues ER stress and reduces inflammation and JEV infection in mice brain. Cytokine.

[34] Elemam NM, Talaat IM, Maghazachi AA (2022). CXCL10 chemokine: a critical player in RNA and DNA viral infections. Viruses.

[35] Li H, Sun Y, Sun L (2022). A teleost CXCL10 is both an immunoregulator and an antimicrobial. Front Immunol.

[36] Mass E, Nimmerjahn F, Kierdorf K, Schlitzer A (2023). Tissue-specific macrophages: how they develop and choreograph tissue biology. Nat Rev Immunol.

[37] Liu J, Zhang X, Cheng Y, Cao X (2021). Dendritic cell migration in inflammation and immunity. Cell Mol Immunol.

[38] Liu Z, Wang H, Li Z, Dress RJ, Zhu Y, Zhang S, De Feo D, Kong WT, Cai P, Shin A, Piot C, Yu J, Gu Y, Zhang M, Gao C, Chen L, Wang H, Vétillard M, Guermonprez P, Kwok I, Ng LG, Chakarov S, Schlitzer A, Becher B, Dutertre CA, Su B, Ginhoux F (2023). Dendritic cell type 3 arises from Ly6C + monocyte-dendritic cell progenitors. Immunity.

[39] Metcalf TU, Wilkinson PA, Cameron MJ, Ghneim K, Chiang C, Wertheimer AM, Hiscott JB, Nikolich-Zugich J, Haddad EK (2017). Human monocyte subsets are transcriptionally and functionally altered in aging in response to pattern recognition receptor agonists. J Immunol.

[40] Tavenier J, Rasmussen LJH, Houlind MB, Andersen AL, Panum I, Andersen O, Petersen J, Langkilde A, Nehlin JO (2020). Alterations of monocyte NF-κB p65/RelA signaling in a cohort of older medical patients, age-matched controls, and healthy young adults. Immun Ageing.

[41] Connors J, Taramangalam B, Cusimano G, Bell MR, Matt SM, Runner K, Gaskill PJ, DeFilippis V, Nikolich-Žugich J, Kutzler MA, Haddad EK (2022). Aging alters antiviral signaling pathways resulting in functional impairment in innate immunity in response to pattern recognition receptor agonists. GeroScience.

[42] Ault R, Dwivedi V, Koivisto E, Nagy J, Miller K, Nagendran K, Chalana I, Pan X, Wang SH, Turner J (2018). Altered monocyte phenotypes but not impaired peripheral T cell immunity may explain susceptibility of the elderly to develop tuberculosis. Exp Gerontol.

[43] Lewis SA, Sureshchandra S, Zulu MZ, Doratt B, Jankeel A, Ibraim IC, Pinski AN, Rhoades NS, Curtis M, Jiang X, Tifrea D, Zaldivar F, Shen W, Edwards RA, Chow D, Cooper D, Amin A, Messaoudi I (2021). Differential dynamics of peripheral immune responses to acute SARS-CoV-2 infection in older adults. Nat Aging.

[44] Groom JR, Luster AD (2011). CXCR3 ligands: redundant, collaborative and antagonistic functions. Immunol Cell Biol.

[45] Wang X, Zhang Y, Wang S, Ni H, Zhao P, Chen G, Xu B, Yuan L (2022). The role of CXCR3 and its ligands in cancer. Front Oncol.

[46] Guo Y, Kasahara S, Jhingran A, Tosini NL, Zhai B, Aufiero MA, Mills KAM, Gjonbalaj M, Espinosa V, Rivera A, Luster AD, Hohl TM (2020). During aspergillus infection, monocyte-derived DCs, neutrophils, and plasmacytoid DCs enhance innate immune defense through CXCR3-dependent crosstalk. Cell Host Microbe.

[47] Chami B, Yeung A, Buckland M, Liu H, Fong M, Tao G, Bao K (2017). CXCR3 plays a critical role for host protection against *Salmonellosis*. Sci Rep.

[48] Ichikawa A, Kuba K, Morita M, Chida S, Tezuka H, Hara H, Sasaki T, Ohteki T, Ranieri VM, dos Santos CC, Kawaoka Y, Akira S, Luster AD, Lu B, Penninger JM, Uhlig S, Slutsky AS, Imai Y (2013). CXCL10-CXCR3 enhances the development of neutrophil-mediated fulminant lung injury of viral and nonviral origin. Am J Respir Crit Care Med.

[49] Flores-Romero H, Ros U, Garcia-Saez AJ (2020). Pore formation in regulated cell death. EMBO J.

[50] D’Cruz AA, Speir M, Bliss-Moreau M, Dietrich S, Wang S, Chen AA, Gavillet M, Al-Obeidi A, Lawlor KE, Vince JE, Kelliher MA, Hakem R, Pasparakis M, Williams DA, Ericsson M, Croker BA (2018). The pseudokinase MLKL activates PAD4-dependent NET formation in necroptotic neutrophils. Sci Signal.

[51] Kitur K, Wachtel S, Brown A, Wickersham M, Paulino F, Peñaloza HF, Soong G, Bueno S, Parker D, Prince A (2016). Necroptosis promotes *Staphylococcus aureus* clearance by inhibiting excessive inflammatory signaling. Cell Rep.

[52] Conos SA, Chen KW, De Nardo D, Hara H, Whitehead L, Núñez G, Masters SL, Murphy JM, Schroder K, Vaux DL, Lawlor KE, Lindqvist LM, Vince JE (2017). Active MLKL triggers the NLRP3 inflammasome in a cell-intrinsic manner. Proc Natl Acad Sci USA.

[53] Peng C, Tu G, Wang J, Wang Y, Wu P, Yu L, Li Z, Yu X (2023). MLKL signaling regulates macrophage polarization in acute pancreatitis through CXCL10. Cell Death Dis.

[54] Kitur K, Parker D, Nieto P, Ahn DS, Cohen TS, Chung S, Wachtel S, Bueno S, Prince A (2015). Toxin-induced necroptosis is a major mechanism of *Staphylococcus aureus* lung damage. PLoS Pathog.

[55] Horst SA, Hoerr V, Beineke A, Kreis C, Tuchscherr L, Kalinka J, Lehne S, Schleicher I, Köhler G, Fuchs T, Raschke MJ, Rohde M, Peters G, Faber C, Löffler B, Medina E (2012). A novel mouse model of *Staphylococcus aureus* chronic osteomyelitis that closely mimics the human infection: an integrated view of disease pathogenesis. Am J Pathol.

[56] Cassat JE, Hammer ND, Campbell JP, Benson MA, Perrien DS, Mrak LN, Smeltzer MS, Torres VJ, Skaar EP (2013). A secreted bacterial protease tailors the *Staphylococcus aureus* virulence repertoire to modulate bone remodeling during osteomyelitis. Cell Host Microbe.

