# Supplementary material for: Age-related dysregulation of CXCL9/10 in monocytes is linked to impaired innate immune responses in a mouse model of Staphylococcus aureus osteomyelitis
Source: Cell Mol Life Sci. 2024 Jul 13;81(1):300. doi: 10.1007/s00018-024-05311-2 (PMC11335224; doi:10.1007/s00018-024-05311-2)
Supplement: Supplementary file 1 — Supplementary Material 1 [file 18_2024_5311_MOESM1_ESM.pdf]

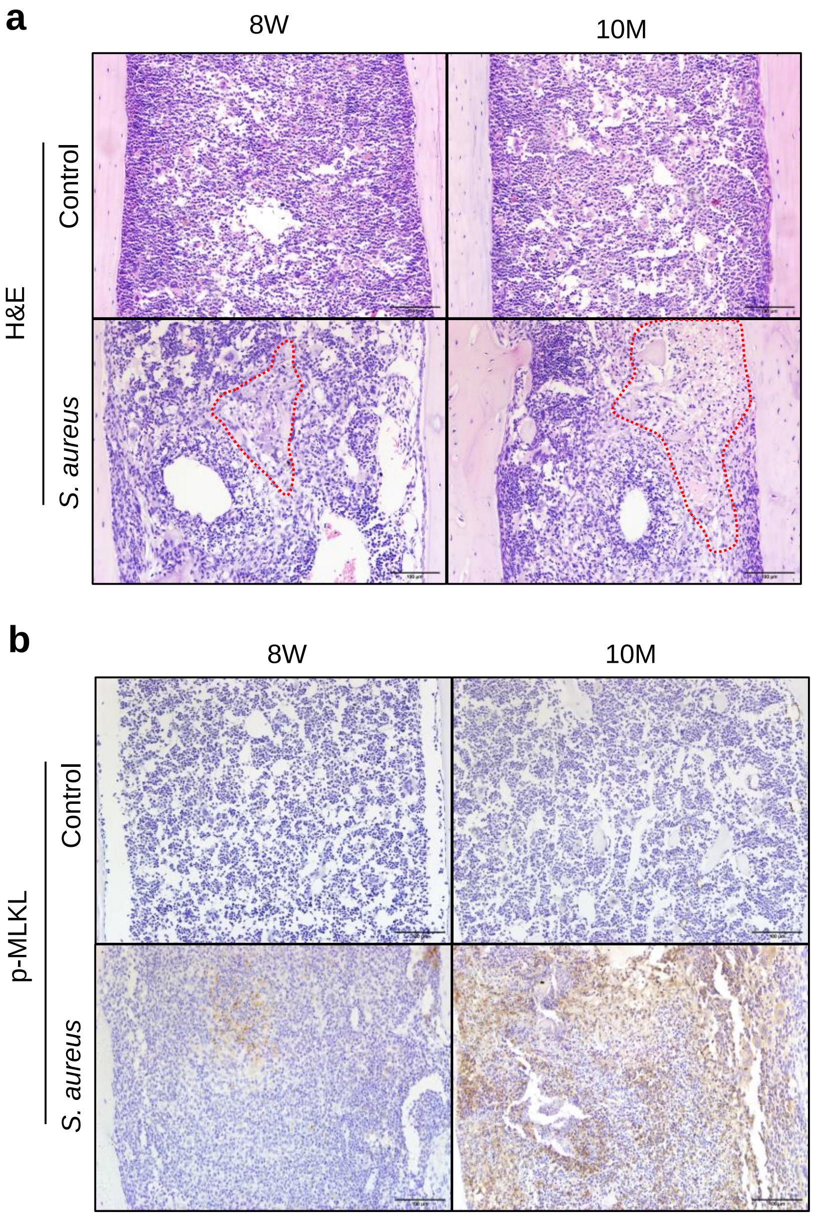

Fig. S1. 10-month-old mice have larger area of necrosis in the *S. aureus*-infected femurs. (a) Representative images of H&E staining under low magnification view. The red dashed area shows necrotic area with accumulation of cell debris. Scale bars: 100 μm. (b) Representative images of immunostaining for phosphorylated mixed lineage domain-like (p-MLKL) under low magnification view. Scale bars: 100 μm.

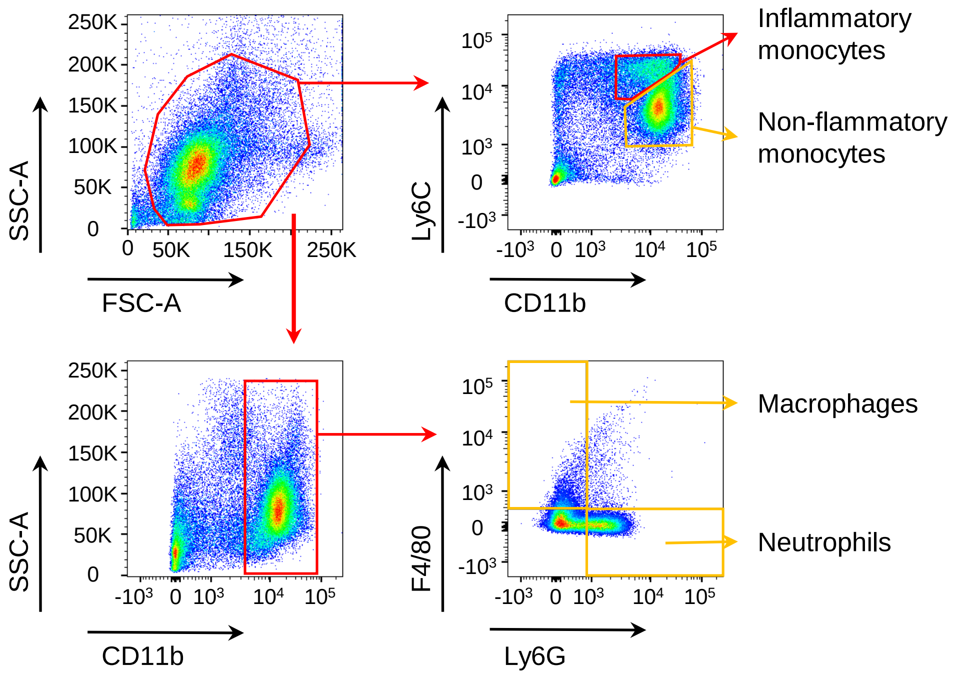

Fig. S2. Dot plots of windows and gating strategy for the identification of inflammatory monocytes ($CD11b^{+}Ly6C^{high}$), non-inflammatory monocytes ($CD11b^{+}Ly6C^{low}$), macrophages ($CD11b^{+}F4/80^{+}Ly6G^{-}$), and neutrophils ($CD11b^{+}Ly6G^{+}F4/80^{-}$).

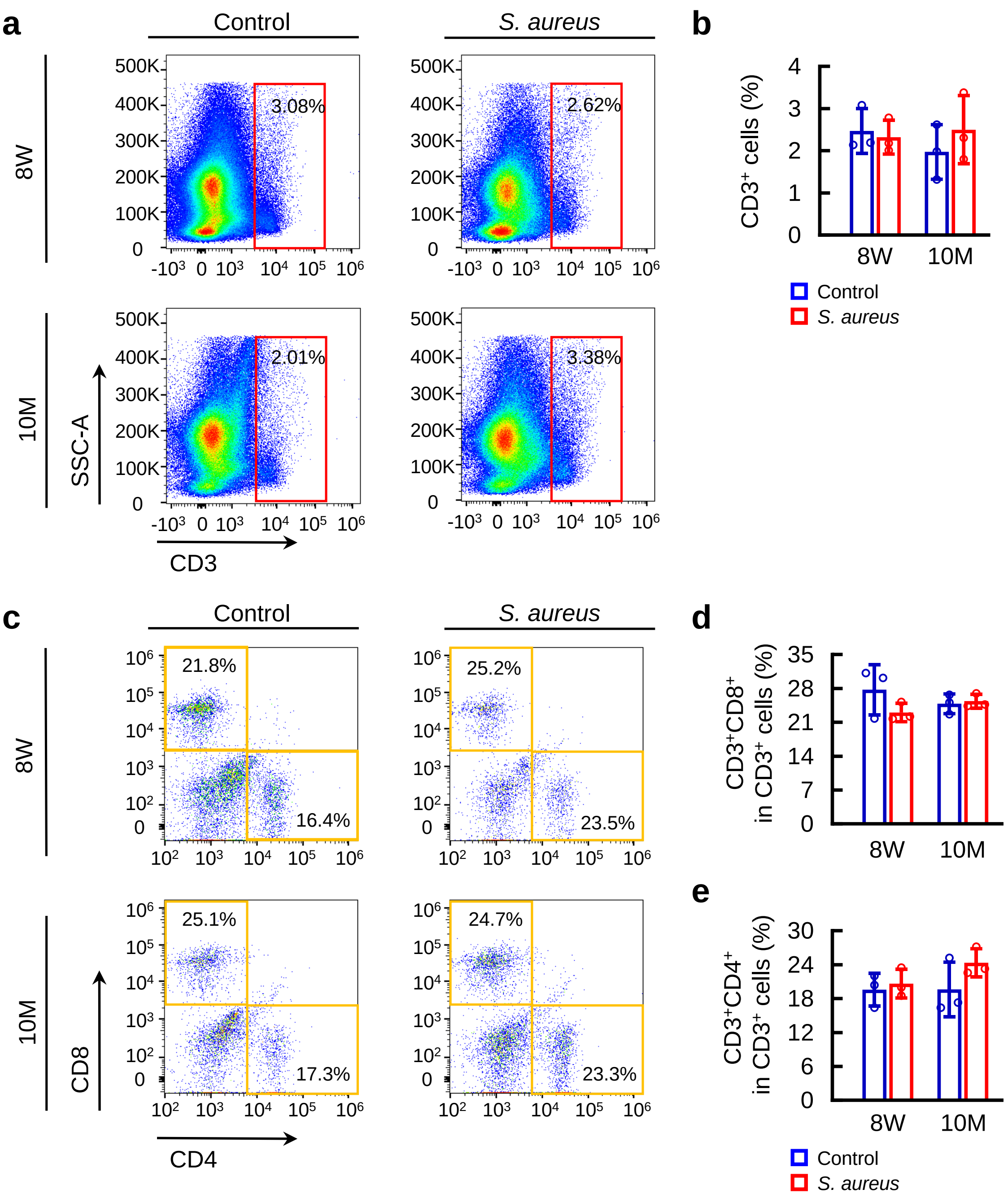

Fig. S3. Changes of $CD3^+$, $CD3^+CD4^+$ and $CD3^+CD8^+$ T cells in mice bone marrow by day 3 post *S. aureus* infection. (a) Representative images of flow cytometry analysis and (b) quantitative analysis of $CD3^+$ cells in total bone marrow cells. (c) Representative images of flow cytometry analysis and quantitative analysis of $CD3^+CD8^+$ cells (d) and $CD3^+CD4^+$ cells (e) in $CD3^+$ cells population.

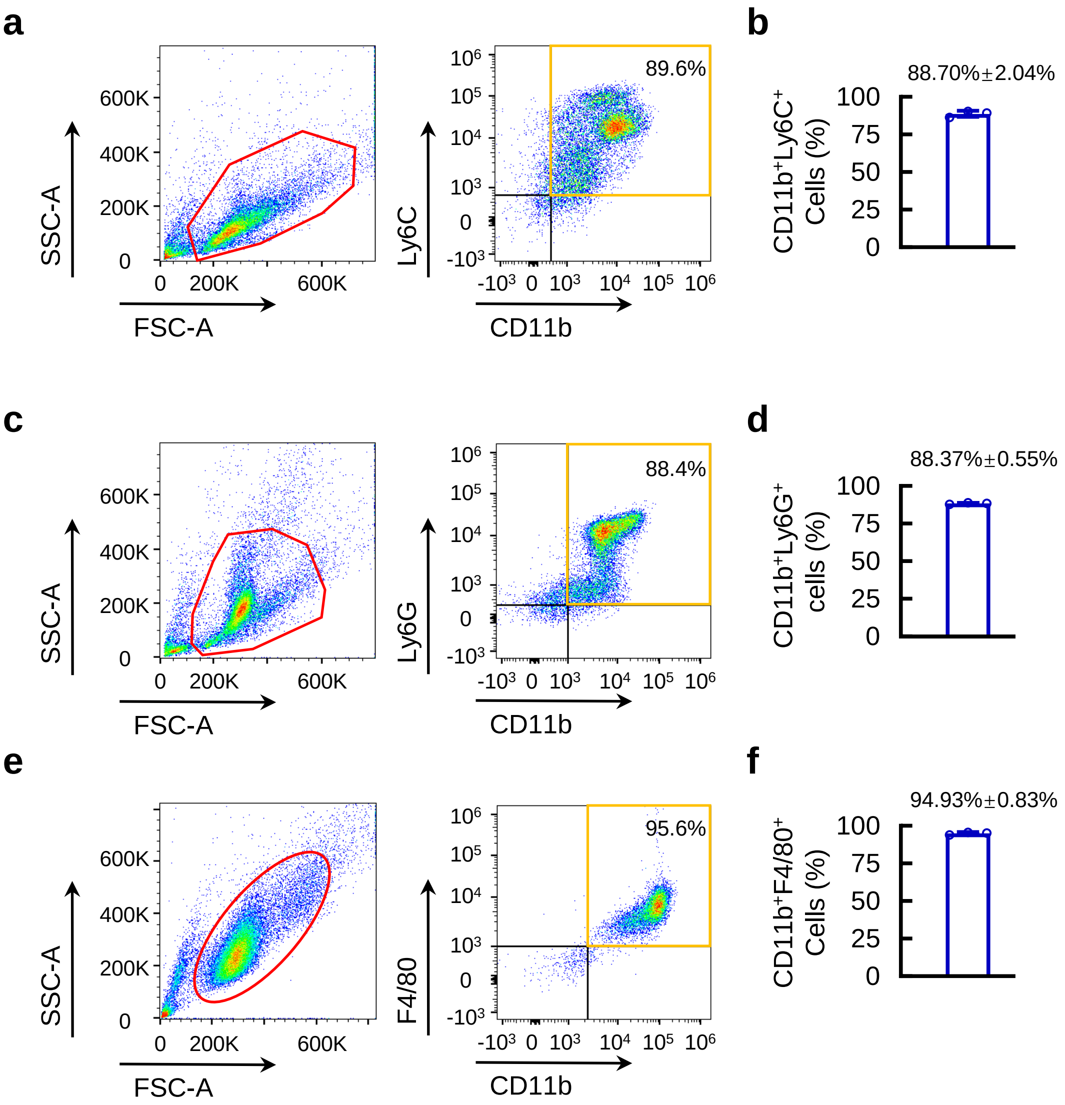

Fig. S4. Flow cytometric analysis of surface markers of monocytes and neutrophils isolated from bone marrow by centrifugation in Percoll, and primary culture of bone marrow-derived macrophages (BMDMs). (a and b) Flow cytometric assay and quantification of $CD11b^{+}Ly6C^{+}$ cells level in collected monocytes from bone marrow. (c and d) Flow cytometric assay and quantification of $CD11b^{+}Ly6G^{+}$ cells level in collected neutrophils from bone marrow. (e and f) Flow cytometric assay and quantification of $CD11b^{+}F4/80^{+}$ cells in primary culture of BMDMs. n = 3/group.

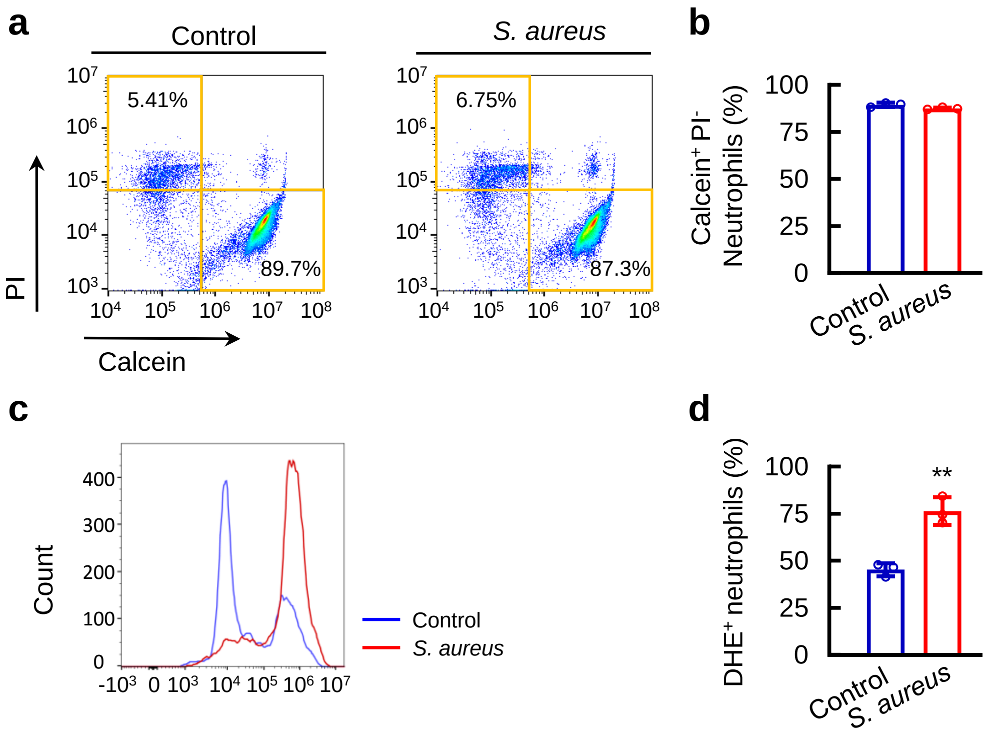

Fig. S5. The viability and ROS production of isolated neutrophils before and after *S. aureus* infection. (a and b) Flow cytometry analysis and quantification of CMFDA^{+}PI^{-} neutrophils. (c and d) Flow cytometry analysis and quantification of reactive oxygen species (ROS) levels probed with dihygroethidium (DHE) in isolated neutrophils. n = 3/group, Student's *t* test, ** $p < 0.01$.

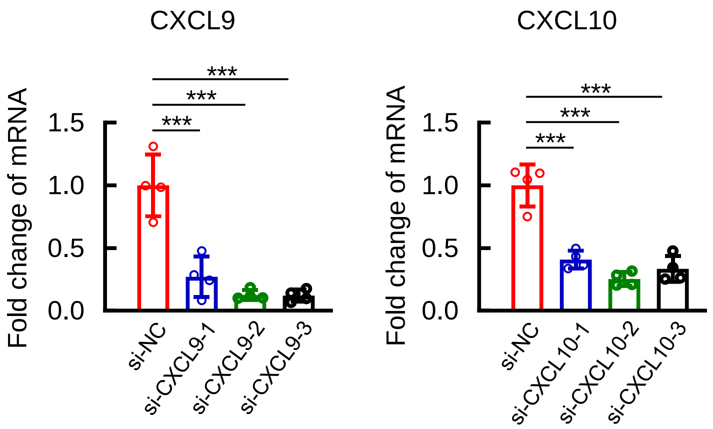

Fig. S6. si-RNA-mediated knockdown of endogenous CXCL9 and CXCL10 expression in primary monocytes. Monocytes were isolated from bone marrow by gradient centrifugation in Percoll, and then transfected with siRNA fragments for CXCL9 and CXCL10, respectively. Total RNA were collected after 24 h and mRNA expression of CXCL9 and CXCL10 were measured by qPCR. n = 4/group, one-way ANOVA with Bonferroni *post hoc* test, *** $p < 0.001$.
